# Prediction of bioluminescent proteins by using sequence-derived features and lineage-specific scheme

**DOI:** 10.1186/s12859-017-1709-6

**Published:** 2017-06-05

**Authors:** Jian Zhang, Haiting Chai, Guifu Yang, Zhiqiang Ma

**Affiliations:** 1 School of Computer Science and Information Technology, Northeast Normal University, Changchun, Jilin Province 130117 People’s Republic of China; 2 School of Computer and Information Technology, Xinyang Normal University, Xinyang, Henan Province 464000 People’s Republic of China

**Keywords:** Bioluminescent proteins, Sequence-derived, Feature analysis, Lineage-specific

## Abstract

**Background:**

Bioluminescent proteins (BLPs) widely exist in many living organisms. As BLPs are featured by the capability of emitting lights, they can be served as biomarkers and easily detected in biomedical research, such as gene expression analysis and signal transduction pathways. Therefore, accurate identification of BLPs is important for disease diagnosis and biomedical engineering. In this paper, we propose a novel accurate sequence-based method named PredBLP (Prediction of BioLuminescent Proteins) to predict BLPs.

**Results:**

We collect a series of sequence-derived features, which have been proved to be involved in the structure and function of BLPs. These features include amino acid composition, dipeptide composition, sequence motifs and physicochemical properties. We further prove that the combination of four types of features outperforms any other combinations or individual features. To remove potential irrelevant or redundant features, we also introduce Fisher Markov Selector together with Sequential Backward Selection strategy to select the optimal feature subsets. Additionally, we design a lineage-specific scheme, which is proved to be more effective than traditional universal approaches.

**Conclusion:**

Experiment on benchmark datasets proves the robustness of PredBLP. We demonstrate that lineage-specific models significantly outperform universal ones. We also test the generalization capability of PredBLP based on independent testing datasets as well as newly deposited BLPs in UniProt. PredBLP is proved to be able to exceed many state-of-art methods. A web server named PredBLP, which implements the proposed method, is free available for academic use.

**Electronic supplementary material:**

The online version of this article (doi:10.1186/s12859-017-1709-6) contains supplementary material, which is available to authorized users.

## Background

Bioluminescence is a special process of chemiluminescence, which is common in many living organisms across the lineages of bacteria, eukaryota and archaea [1]. Bioluminescent proteins (BLPs), with the capability of emitting light by converting chemical energy to light energy, play a critical role in bioluminescence [2, 3]. Employed as highly sensitive labels, they are enormously useful in non-invasive in-vivo biomedical research, such as gene expression analyses [4] and signal transduction pathways [5]. Since BLPs can be easily detected, they are widely used in bioluminescence imaging (tagging biological entities or process), as biosensors for environmental contaminants, and as detectors to map neuronal circuits [6]. Particularly, BLPs can be used for non-invasive analyses of molecular functions in living cells and organisms. With the help of bioluminescence microscopy, scientists can trace and monitor the chemical reaction by quantifying the photon emission of BLPs (such as luciferase) [7]. The quantified visible light provides clues about the location and status of BLPs implanted into tumors or tissues.

Bioluminescence imaging and biosensors are featured by its capability of providing high-sensitive identification of BLPs. However, these methods all suffer several potential problems, which affect the performance of the detection. First, BLPs are sensitive to the microenvironment [8]. For instance, *D*-Luciferin exhibits the peak spectrum in green region in acidic solution while in red region at basic pH [9]. Second, the vivo organisms largely scatter or absorb the majority regions of the spectrum. Although low temperature can reduce thermal noise, it might also kill the tissues as well as the BLPs. Third, it is difficult to detect light-emitting which is produced inside a living animal without harming its skin. Fourth, light emission is the most significant factor. However, for most bioluminescence signals, they are too weak to detect. Additionally, the filtering to excitation light might affect the corresponding emission light [10]. As a result, the biophysical or biochemical experiments can be benefit from the computational methods which process the characteristic of predicting large amount of data accurately and effectively.

Recent years have witnessed a number of computational methods for predicting BLPs. The earliest study in recognizing BLPs based on computational methods can be traced back to 2011 when Kandaswamy et al. used 544 physicochemical properties and support vector machine to predict BLPs [11]. They also built the first sequence-based predictor named BLProt. Soon after that, Zhao et al. proposed an improved method named BLPre by using evolutionary profiles represented by position specific scoring matrices to construct feature vector [12]. Fan et al. adopted the concept of pseudo amino acid composition to represent proteins and achieved a good prediction quality [13]. Huang et al. introduced the knowledge acquisition method in characterizing BLPs and the evolutionary fuzzy classifier to build prediction model [14]. They also proposed a scoring card method to estimate the propensity scores of dipeptides and amino acids as well as design prediction models [15]. Nath et al. adopted oversampling technique and unsupervised K-means algorithm for predicting BLPs [16].

In summary, these methods provide important clues in this field. Some of them provide web servers or programs. These prediction tools help biologists to fast predict potential BLPs and promote the development of this field. However, as far as we have concerned, there are two aspects that need to be further investigated. First, most of these studies used various types of features to encode the proteins (or the samples). However, they lacked detailed analyses or descriptions of the features. That is, it is uncertain about the discrimination capability of these features. Second*,* most of these studies only considered general BLPs. In other words, they didn’t consider the differences across different lineages of BLPs. Actually, based on our research, these existed differences are valuable for deep investigation. They are expected to further promote the accuracy of the prediction models. However, it has not yet received enough attention.

Motivated by the above-mentioned two drawbacks, in this study, we focus on the challenge of proposing a novel accurate predictor for identifying BLPs based on sequence-derived features. We collect and compile four new datasets (one general and three lineage-specific datasets), which contain non-redundant BLPs and non-BLPs. Next, a series of sequence-derived features, which have been proved to be involved in BLPs, are mathematically computed to encode the proteins. Detailed analyses are performed to empirically show the differences between BLPs and non-BLPs, especially across lineage-specific BLPs. Then, these differences are used to discriminate BLPs against non-BLPs. For the convenience of biology researchers, our method has been implemented as a user-friendly web server named PredBLP (Prediction of BioLuminescent Proteins), which is free available at http://www.inforstation.com/PredBLP/.

## Methods

### Datasets

In this work, we construct four datasets, which include one general and three lineage-specific datasets, for the investigation of BLPs. The three considered lineages include bacteria, eukaryota and archaea. All these datasets are compiled from 17,403 collected BLPs from UniProt (Jul. 2016) [17]. Since the existence of homologous would lead to the bias of the modeling and predicting processes, we further use BLASTClust [18] to cluster all these proteins with a cut-off of 30%. We choose BLASTClust because it is capable of clustering sequences with low similarity as well as long sequences. Next, we randomly pick one protein from each cluster as the representative. Finally, we obtain 863 BLPs (positive samples). Among these BLPs, 748 belong to bacteria, 70 belong to eukaryota and 45 belong to archaea. Additionally, we also collect 7093 non-redundant non-BLPs (negative samples) to construct the negative samples. Among them, 4919, 1426 and 748 proteins are affiliated with bacteria, eukaryota and archaea respectively. We randomly pick 80% of positives and equal number of negatives in each dataset for balanced training. The rest samples are used for independent testing. Detailed information of these newly compiled datasets can be found in our web server.

To fairly compare our proposed method with previous studies [11–13, 15, 16, 19], we also introduce Kandaswamy’s [11] training dataset. The BLPs in Kandaswamy’s training dataset were selected from Pfam database [20]. Then, they used CD-HIT [21] to remove redundant proteins with more than 40% sequence similarity.

### The construction of feature vector

#### The features of amino acid composition

As the principal fundamental elements of the proteins, amino acid composition (AAC) provides useful clues in protein structure and function. The features of AAC are widely used in bioinformatics [22–24]. In this work, the features of AAC for each type of BLPs, including the general BLPs and three lineages of BLPs, are calculated by:

1 $$ {f}_{AA C}(i)=\frac{AA_i}{L} $$

where *AA*_*i*_(*i* ∈ {1, 2, 3,  … , 20}) represents *i*-th type of amino acids, and *L* indicates the length of the query protein. Finally, we quantify the composition of 20 amino acids in the query protein.

#### The features of dipeptide composition

Previous studies have proved that dipeptide composition (DC) plays important roles in protein structure and function, such as vivo activity and protein thermo stability [25]. Hereby, the features of DC then can be formulated as:

2 $$ {f}_{DC}\left( i, j\right)=\frac{\sum_{n=1}^{L-1}{AA}_n{AA}_{n+1}\to {AA}_i{AA}_j}{L-1} $$

where *AA*_*i*_ *AA*_*j*_(*i*, *j* ∈ {1, 2, 3,  … , 20}) represents 400 types of dipeptide, *n* indicates the position of *n*-th residues in the query protein with the length of *L* residues. *AA*_*n*_ *AA*_*n* + 1_ → *AA*_*i*_ *AA*_*j*_ denotes the dipeptide *AA*_*n*_ *AA*_*n* + 1_ in the query protein is same as *AA*_*i*_ *AA*_*j*_ in the 400 dipeptides. *f*_*DC*_(*i*, *j*) quantifies the frequencies of dipeptides using a straightforward statistical approach.

#### The features of sequence motifs

**Fig. 1:**
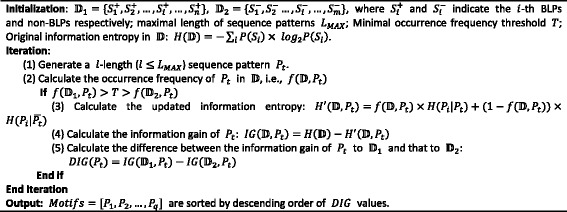
The pseudo-code of the calculation of motifs

Sequence motifs (MTF) in protein sequences always indicate the conserved regions [26]. Although many similarities for proteins in the same family may disappear after long-standing evolution, some inherited attributes still exist because they are functionally or structurally related signals [27]. These signals help to control the cellular localization regions and corresponding biochemical functions [28]. Thus, in this study, we introduce information theory to compute the features of MTF that are more favorable to BLPs against non-BLPs. We first calculate the original information entropy of BLPs and non-BLPs. Then, we iteratively generate a *l*-length pattern *P* from “*AXA*” to “*VXV*” (“*X*” denote random amino acid(s)). For each pattern *P*, we calculate its occurrence frequencies in BLPs and non-BLPs. If its frequency in BLPs is larger than the minimal preset occurrence frequency threshold T (in this study, we preset *T* = 10%), we use this pattern *P* to reclassify samples and calculate the updated information entropy. Then, we compare the original information entropy with the updated one, and generate corresponding information gains of the considered *P*. Next, we calculate the difference of these two information gains (DIG). The higher the difference is, the more discriminatory the pattern is. The pseudo-code of the aforementioned procedure is shown in Fig. 1.

In this work, we choose the top 10 motifs which are sorted by descending order of DIG values. Next, we create a 10-dimensional binary vector to denote whether or not a query protein contains the considered 10 motifs. We use the number ‘1’ to represent the positive and ‘0’ to indicate the negative.

#### The features of physicochemical properties

Since amino acids serve as building blocks of proteins, the physiochemical properties (PCP) of amino acids influence the microscopic environment, which includes surface motions, energy, and dynamics [23, 27, 29]. In this part, we further investigate several properties related to BLPs.

Alipour et al. found that the insertion and substitution of positively-charged residues effect the light shift mechanism [30]. Li et al. proved that the hydrophobicity in active site determines the activity of BLPs [31]. Moradi et al. pointed out that the change in polarity of the emitter site of BLPs lead to the modulation of the bioluminescence color [32, 33]. Particularly, the movement of flexible loop in BLPs usually concomitantly changes the polarity of the emitter site [32]. For instance, if a bulge appears in a flexible loop, the emission lights shifts color from green to red. With the help of energy acceptors, the energy transfer changes the bioluminescence intensity as well as effects the spectral shifts [34, 35]. Silva et al. stated that the increase in polarity causes a decrease in the emission energies. They also provide the evidences that the change of solvent and pH affect the structural and electronic properties of BLPs [36]. Considering this, we collect the physicochemical properties, which include hydrophobicity [31], hydrophilicity [37], polarity [38], polarizability [39], transfer free energy [40], solvent contact area [41], positively-charge [42], flexibility [43] and protein kinase A [44]. Given a query protein, its features of PCP are calculated as follows:

3 $$ {f}_{PCP}(i)=\frac{PCP_i- \min \left(\frac{1}{L}\sum_{j=1}^L{PCP}_{i, j}\right)}{ \max \left(\frac{1}{L}\sum_{j=1}^L{PCP}_{i, j}\right)- \min \left(\frac{1}{L}\sum_{j=1}^L{PCP}_{i, j}\right)} $$

where *i* representes the *i*-th PCP and *j* indicates the *j*-th amino acid in the query protein with the length of *L* residues. Detailed information of these properties is provided in Additional file 1: Table A1.

### Feature selection strategy

The combination of various types of features could provide more useful information in constructing a model [22]. However, the existence of irrelevant features (noisy features) or redundant features may potentially deteriorate the prediction quality of the predictor. In view of this, we adopt Fisher-Markov Selector [45] together with Sequential Backward Selection [46] to perform feature selection. The Fisher-Markov Selector is a typical filter method. It uses Markov random fields to achieve the exact global optimization in calculating the correlation coefficients between features and labels. The output of Fisher-Markov Selector is a list of ranked features according to the calculated coefficients. Next, Sequential Backward Selection strategy is introduced by iteratively removing the least irrelevant features. A feature will never be considered once it is eliminated. The iteration stops until the elimination of features cannot achieve better results. At that time, the remaining features construct the optimal feature subsets.

### Model construction and performance evaluation

**Fig. 2:**
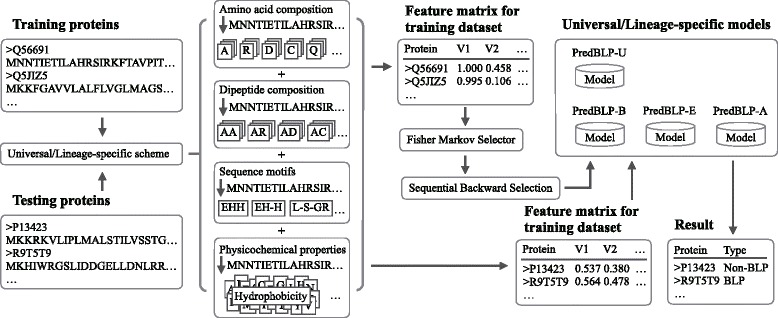
The flowchart of the proposed method

Support vector machine (SVM) [47] has been proved to be a powerful machine learning algorithms [48]. It is widely used to construct prediction model in predicting protein structures and functions [12, 27, 49]. In this study, we use LIBSVM (version 3.20) [50] to train model and perform the prediction. The radial biases function is used as the kernel function and the grid search is adopted to find the optimal parameters and optimize SVM model. Shown in Fig. 2 is the flowchart of our proposed method.

We assess our method using two statistical methods, namely *k*-fold cross-validation and the independent test. For the *k*-fold cross-validation, the samples in training dataset are divided into *k* equal subsets. In each iteration, *k*-1 subsets are used as training data to train the model and the remaining one is used as the validation data to test the model. This procedure repeats *k* times, and the final performance is measured by averaging the results of *k* iterations. For the independent test, the samples in the testing dataset are independent from those in the training dataset. The model that trained in the training dataset is used to predict testing datasets. In this study, the binary-based criteria, including accuracy, sensitivity, specificity and Matthew’s Correlation Coefficients (MCC) are used to evaluate the methods which output binary predictions.

4 $$ Accuracy=\frac{TP+ TN}{TP+ FP+ TN+ FN} $$

5 $$ Sensitivity=\frac{TP}{TP+ FN} $$

6 $$ Specificity=\frac{TN}{TN+ FP} $$

7 $$ MCC=\frac{TP\times TN- FP\times FN}{\sqrt{\left( TP+ FP\right)\left( TN+ FN\right)\left( TP+ FN\right)\left( TN+ FP\right)}} $$

where TP, TN, FP, FN indicate the true positives (correctly predicted as BLPs), true negatives (correctly predicted as non-BLPs), false positives (incorrectly predicted as BLPs) and false negatives (incorrectly predicted as non-BLPs), respectively. In the case that the prediction probability is available, we introduce score-based metric for assessing the methods that produce predicted propensities. Similar to other methods, we also report AUC values, which stands for the area under the ROC (Receiver Operating Characteristic) curve.

## Results and discussion

### The characteristics of the extracted features

In this work, we construct the feature space based on multiple types of features including AAC, DC, MTF and PCP. Before putting them into operation, we examine their characteristics on BLPs and non-BLPs.

**Fig. 3:**
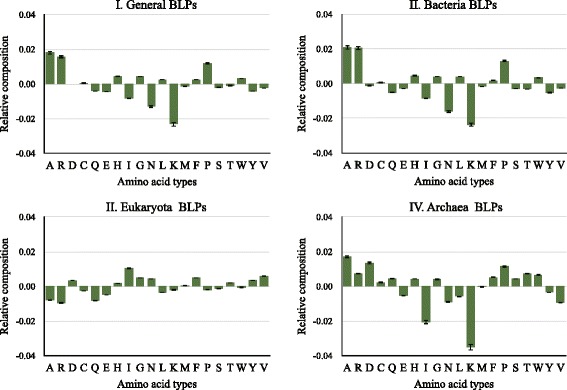
The relative amino acid composition of BLPs against non-BLPs on four datasets

To investigate the amino acid preference of BLPs, we calculate the features of AAC for BLPs and non-BLPs respectively. Illustrated in Fig. 3 is the relative amino acid composition of BLPs against non-BLPs in four datasets. Generally, compared with the non-BLPs, BLPs are enriched with charged residues. This phenomenon keeps consistent in bacteria and archaea BLPs. Moreover, bacteria BLPs are enriched with buried and depleted with acidic amino acids; eukaryota BLPs are enriched with aliphatic and aromatic amino acids; and archaea BLPs are enriched with acyclic and cyclic amino acids and depleted with aliphatic amino acids. We also find that the relative differences on eukaryota BLPs against non-BLPs are relative lower than those on bacteria and archaea BLPs. Detailed data of their values is provided in Additional file 1: Table A2. We empirically demonstrate that amino acid compositions with relative difference higher than 0.25% are discriminatory.

**Fig. 4:**
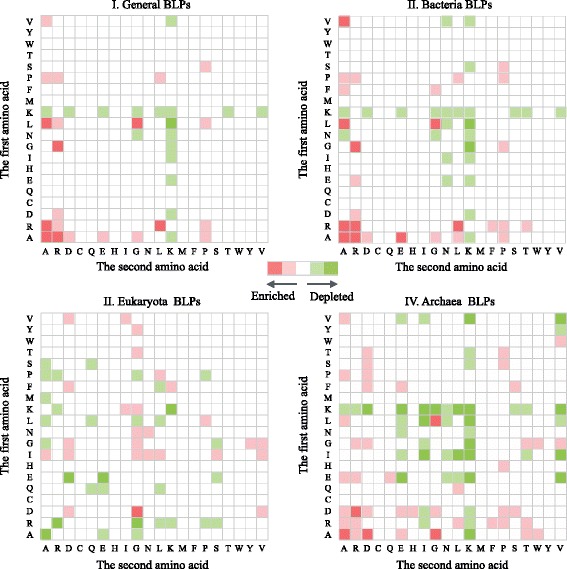
The relative dipeptide composition of BLPs against that of non-BLPs in four datasets. The *x*-axis indicates the amino acids which are cleaved on the C-terminal side; while *y*-axis stands for the N-terminal side. Detailed data of their values is provided in Additional file 1: Tables A3-A6

Illustrated in Fig. 4 is the relative dipeptide composition of BLPs against that of non-BLPs in four datasets. Red block indicates the discriminatory enriched dipeptides, while green one represents the opposite. The deeper the color is, the more significant the enrichment/depletion is. Generally, BLPs show high preference with A-, R-, P- and G-related dipeptide, which keeps consistent with those in bacteria and archaea BLPs. For general BLPs, the ‘A-A’, ‘A-R’, ‘R-A’ and ‘R-L’ dipeptides show over-represented than normal level. In eukaryota BLPs, I- and G-related dipeptides are more favored. Moreover, the K-related dipeptides are under-represented on both C-terminal and N-terminal side in general BLPs and three lineages of BLPs. The motifs can also be used to further discriminate various lineages.

Table 1 lists the top 10 selected motifs according to the descending order of DIG values, which mathematically indicate the relative distinguish capability of various motifs. The higher the DIG value is, the more quantified difference the motif exists in BLPs against non-BLPs.

**Table 1:** Selected top 10 motifs according to the descending order of DIG values

Lineage: general		Lineage: bacteria		Lineage: eukaryota		Lineage: archaea	
Motif	DIG	Motif	DIG	Motif	DIG	Motif	DIG
EHH	0.669	EHH	0.693	G-T-G-P	0.617	DGW	0.809
EH-H	0.627	LS-GR	0.675	SG-T-G	0.574	G-GW	0.800
L-S-GR	0.608	EH-H	0.644	GM-E	0.563	A-TLD	0.779
E-HH	0.607	L-S-GR	0.629	G-M-E	0.518	A-A-T-D	0.745
LS-G-R	0.588	E-HH	0.624	FVE	0.505	D-W-P	0.741
L-G-GR	0.579	L-G-GR	0.621	TGD	0.494	A-T-LD	0.733
E-H-H	0.561	LS-G-R	0.608	FD-I	0.489	A-TL-D	0.726
S-G-G-R	0.536	S-G-G-R	0.587	D-GY	0.479	D-GW	0.726
A-A-T-R	0.519	E-H-H	0.565	F-YG	0.461	GFD	0.704
L-S-G-R	0.515	A-A-T-R	0.542	F-M-G	0.460	DG-W	0.704

**Fig. 5:**
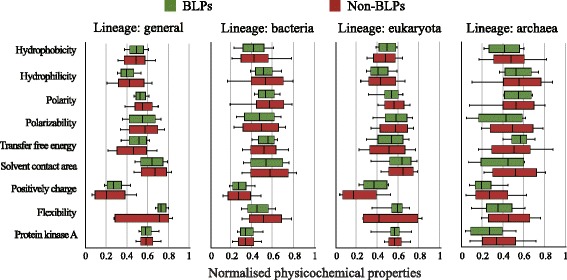
Basal levels of selected physicochemical and biological properties in four datasets. Midline, box boundaries, and whiskers indicate median, quartiles, and 10th and 90th percentiles. The x-axis indicates the normalized values; and y-axis stands for twelve properties. In this work, a physicochemical property is empirically regarded to be discriminatory provided that the overlap of two boxes is less than 80% of either box

Illustrated in Fig. 5 are the median based box plots for the considered nine physicochemical properties. We notice that the variability of these properties in BLPs is overall much lower than that for non-BLPs. For instance, the values of polarity, positively charge and flexibility in general BLPs are less volatile than those in non-BLPs. This phenomenon keeps consensus in three lineage-specific datasets. Additionally, eukaryota BLPs are more flexible than bacteria and archaea BLPs. O’Brien et al. pointed out that broad dynamic range and stable signals in eukaryota BLPs are the reasons for the increased flexibility [51].

### The study on the direct feature combination

From the perspective of machine learning, the combination of various types of features usually produces better performance than individual features do. Therefore, we test the effectiveness of individual features as well as different combinations of features. Hereby, we adopt five-fold cross-validation on the training dataset. The final results are reported by calculating the average value and standard deviations of five experiments.

As shown in Table 2, four types of features all give out promising prediction results. The features of DC produce the highest MCC (0.650 ± 0.029) and AUC (0.830 ± 0.016) among four individual features. Generally, the combination of two types of features shows higher accuracy of prediction, which is also true for three when compared with two. The combination of four types of features achieves the best performance with MCC = 0.676 ± 0.010 and AUC = 0.850 ± 0.006. This experiment proves the effectiveness of proposed features, and further indicates that the combination of different types of features can produce a promising result. Similar experiments on the other three training sets are provided in Additional file 1: Table A7.

**Table 2:** The experimental results of various individual and combinative features on the training set for general BLPs

Type	Feature	Sensitivity	Specificity	Accuracy	MCC	AUC
Individual	AAC^a^	0.729 ± 0.029	0.806 ± 0.023	0.767 ± 0.020	0.537 ± 0.039	0.802 ± 0.012
	DC^b^	0.791 ± 0.017	0.857 ± 0.028	0.824 ± 0.014	0.650 ± 0.029	0.830 ± 0.016
	MTF^c^	0.313 ± 0.017	0.942 ± 0.012	0.628 ± 0.008	0.328 ± 0.018	0.653 ± 0.010
	PCP^d^	0.452 ± 0.010	0.910 ± 0.026	0.681 ± 0.010	0.408 ± 0.029	0.763 ± 0.014
Combinative	AAC + DC	0.799 ± 0.015	0.862 ± 0.026	0.830 ± 0.008	0.663 ± 0.018	0.841 ± 0.012
	AAC + MTF	0.764 ± 0.016	0.801 ± 0.021	0.783 ± 0.005	0.566 ± 0.011	0.810 ± 0.007
	AAC + PCP	0.728 ± 0.013	0.809 ± 0.013	0.768 ± 0.007	0.538 ± 0.015	0.813 ± 0.008
	DC + MTF	0.799 ± 0.014	0.854 ± 0.008	0.826 ± 0.008	0.653 ± 0.015	0.836 ± 0.005
	DC + PCP	0.775 ± 0.014	0.878 ± 0.020	0.827 ± 0.004	0.658 ± 0.009	0.841 ± 0.006
	MTF + PCP	0.477 ± 0.010	0.917 ± 0.016	0.697 ± 0.004	0.440 ± 0.014	0.764 ± 0.020
	AAC + DC + MTF	0.772 ± 0.007	0.888 ± 0.011	0.830 ± 0.008	0.665 ± 0.016	0.842 ± 0.006
	AAC + DC + PCP	0.780 ± 0.007	0.880 ± 0.016	0.830 ± 0.009	0.663 ± 0.019	0.845 ± 0.009
	AAC + MTF + PCP	0.742 ± 0.011	0.793 ± 0.004	0.767 ± 0.005	0.536 ± 0.010	0.816 ± 0.004
	DC + MTF + PCP	0.775 ± 0.014	0.886 ± 0.025	0.830 ± 0.011	0.665 ± 0.023	0.845 ± 0.014
	AAC + DC + MTF + PCP	0.770 ± 0.010	0.894 ± 0.014	0.836 ± 0.004	0.676 ± 0.010	0.850 ± 0.006

The results are reported by maximizing the MCC value of prediction on the corresponding dataset over five-fold cross-validation. ^a^ indicates the features of amino acid composition; ^b^ stands for the features of dipeptide composition; ^c^ is the features of motifs; ^d^ represents the features of physicochemical properties

### The performance of feature selection scheme

Although the combination of different types of features can improve the prediction accuracy, some noisy data would be also added in the feature vector. Here, we decide to select the optimal feature subset. As stated in Feature selection strategy, we first use Fisher-Markov Selector to calculate correlation coefficients of different features (Additional file 1: Figure A1). Next, we adopt Sequential Backward Selection strategy to select the optimal classifier and corresponding optimal feature subset. Finally, we obtain 199 features on the general BLPs dataset, and 174, 204 and 129 features on three lineages-specific BLPs datasets respectively (Table 3). Based on the optimal feature subset, the classifier on general BLPs achieves the MCC of 0.698 ± 0.018 and AUC of 0.883 ± 0.007, which are 0.022 (or 3.3%) and 0.033 (or 3.9%) higher than that based on the complete features. Three lineages-specific models also show similar increase in the prediction accuracy.

**Table 3:** The performance of optimum feature subsets on four training sets using five-fold cross-validation

Lineage	Number	Sensitivity	Specificity	Accuracy	MCC	AUC
General	199	0.732 ± 0.010	0.949 ± 0.022	0.841 ± 0.006	0.698 ± 0.018	0.883 ± 0.007
Bacteria	174	0.832 ± 0.012	0.943 ± 0.016	0.888 ± 0.006	0.780 ± 0.013	0.920 ± 0.010
Eukaryota	204	0.667 ± 0.053	0.833 ± 0.053	0.750 ± 0.026	0.510 ± 0.054	0.806 ± 0.015
Archaea	129	0.825 ± 0.061	0.900 ± 0.094	0.863 ± 0.047	0.733 ± 0.095	0.917 ± 0.019

The results are reported by maximizing the MCC value of prediction on the corresponding dataset

In section “The characteristics of the extracted features”, we detailedly characterize the intrinsic differences across general BLPs, bacteria BLPs, eukaryota BLPs, archaea BLPs and non-BLPs. After that, we perform the feature selection. Then the calculated optimal feature subset (Additional file 1: Table A8) is used to train the model. To check whether these differences are still kept after the feature selection, we further investigate the composition of the optimal feature subset.

**Fig. 6:**
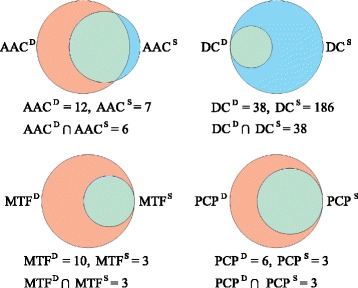
Venn diagrams of the overlap (*green zone*) between the discriminatory (*orange pie*) and selected useful features (*blue pie*) in the optimal subset for each type of features. D indicates the discriminatory features and s stands for selected useful features

Figure 6 shows the overlap between the discriminatory and selected useful features, respectively. Among the 12 discriminatory features within AAC, 6 (or 50%) are selected in the optimal feature subset. More importantly, it occupies 85.7% (6/7 = 85.7%) of the selected AAC. The overlap is even higher for the features of DC, MTF and PCP. We notice that all discriminatory features are successfully selected during the feature selection procedure. This suggests that the calculated differences could be valuable in distinguishing BLPs from non-BLPs. The Venn diagrams for three lineage-specific optimal subsets are illustrated in Additional file 1: Figure A2. We see the similar results from Additional file 1: Figure A2. Mathematically, the existent differences in the features help the classifier to discriminate samples. That’s why the fraction of overlap is as high as expected. In follow-up experiments, we use the optimal feature subset to train universal model or lineage-specific ones.

### Comparison of lineage-specific scheme with traditional universal approach

As stated in section “The characteristics of the extracted features”, we find that BLPs in different lineages have various attributes on our considered features. These various attributes can be used to further improve the prediction performance by introducing lineage-specific scheme. Considering this, we design three lineage-specific classifiers in addition to traditional universal one. In this section, we evaluate the effectiveness of this scheme.

Table 4 compares the performance between lineage-specific models (PredBLP-B, PredBLP-E and PredBLP-A) and universal models (PredBLP-U) on three training datasets. The lineage-specific models improve the average AUC values of 0.048 ~ 0.136 (or 5.5% ~ 20.3%) when compared with universal ones. We also notice that this scheme performs the best for eukaryota BLPs, which corresponds to the investigation of the differences across various lineages. To sum up, the experimental results demonstrate the effectiveness of the lineage-specific scheme.

**Table 4:** Comparison of lineage-specific models with traditional universal models on three training sets using five-fold cross-validation

Lineage	Model	Sensitivity	Specificity	Accuracy	MCC	AUC
Bacteria	PredBLP-U	0.790 ± 0.010	0.918 ± 0.014	0.854 ± 0.003	0.714 ± 0.007	0.872 ± 0.006
	PredBLP-B	0.832 ± 0.012	0.943 ± 0.016	0.888 ± 0.006	0.780 ± 0.013	0.920 ± 0.010
Eukaryota	PredBLP-U	0.417 ± 0.053	0.883 ± 0.041	0.650 ± 0.033	0.340 ± 0.075	0.670 ± 0.017
	PredBLP-E	0.667 ± 0.053	0.833 ± 0.053	0.750 ± 0.026	0.510 ± 0.054	0.806 ± 0.015
Archaea	PredBLP-U	0.750 ± 0.079	0.875 ± 0.079	0.813 ± 0.040	0.637 ± 0.081	0.868 ± 0.016
	PredBLP-A	0.825 ± 0.061	0.900 ± 0.094	0.863 ± 0.047	0.733 ± 0.095	0.917 ± 0.019

The results are reported by maximizing the MCC values of prediction on the corresponding dataset over five-fold cross-validation. PredBLP-U stands for the universal model of the proposed PredBLP predictor. PredBLP-B, PredBLP-E and PredBLP-A indicate three lineage-specific models (i.e. bacteria-, eukaryota- and archaea- specific model) respectively

### Comparison with other methods on Kandaswamy’s training dataset

To test the robustness of our method as well as perform fair evaluation with previous studies [11, 12, 15, 16, 19], we also introduce Kandaswamy’s training dataset [11]. Next, we compare our method with BLProt [11], BLPre [12], Fan’s method [13], SCMBLP [15], BLKnn [19] and Nath’s method [16]. The results of these methods on Kandaswamy’s training dataset are directly obtained from their reports. Since the Kandaswamy’s training dataset does not particularly annotate the lineage of BLPs, we use the traditional universal approach to build the prediction model (PredBLP-U). Since all these methods use different way to under-sample Kandaswamy’s dataset, the potential bias may exist in the process of sampling. Considering this, we repeat the under-sampling procedure for 10 times and report the corresponding average results.

As shown in Table 5, all methods produce good results with sensitivity > 0.7, specificity > 0.9 and AUC > 0.85. We notice that all methods achieve good predictions. It should be noted that Kandaswamy et al. used CD-HIT [18] to remove redundant proteins with more than 40% sequence similarity. Actually, a common rule is that two sequences are homologous if they are more than 30% identical over their entire lengths [52]. The existence of homology proteins results in a relative easy dataset for each method. Our method also shows promising results with sensitivity = 0.912 ± 0.014 and specificity = 0.962 ± 0.017. PredBLP-U yields an AUC value of 0.968 ± 0.009, which is slightly lower than that of Nath’s method. Among these considered predictors, Nath’s method gives out the highest AUC of 0.991. Our PredBLP-U achieves the highest MCC value (0.849 ± 0.019) and second highest AUC value (0.968 ± 0.009).

**Table 5:** Comparison of the proposed PredBLP-U with previous methods on Kandaswamy’s training dataset

Method	Sensitivity	Specificity	Accuracy	MCC	AUC
BLProt [11]	0.745	0.842	0.801	0.590	0.870
BLPre [12]	0.793	0.910	0.852	N/A	0.920
Fan’s method [13]	0.883	0.927	0.905	0.810	0.950
SCMBLP [15]	0.897	0.920	0.908	N/A	N/A
BLKnn [19]	0.749	0.955	0.852	0.719	N/A
Nath’s method [16]	0.964	0.942	0.954	N/A	0.991
PredBLP-U	0.912 ± 0.014	0.962 ± 0.017	0.937 ± 0.009	0.875 ± 0.018	0.968 ± 0.009

### Comparison with other predictors on independent testing datasets

In order to test the generalization capability of our method, we further test PredBLP on four independent testing sets including general BLPs and three lineages of BLPs. Here, we compare our method with BLProt [11] and SCMBLP [15] because the rest predictors were either no longer maintained or unavailable. Meanwhile, we also test the universal model and lineage-specific models on three lineages of BLPs. First, we random picked 80% BLPs and 80% non-BLPs from our independent dataset. Next, we use these proteins to evaluate BLProt and SCMBLP. We repeat this procedure for 10 times to avoid potential bias in under sampling. Finally, we calculate the statistic differences of MCC values between among the stat-of-art predictors.

Table 6 summarizes the prediction results of stat-of-art predictors on independent datasets. Since BLProt and SCMBLP were all constructed based on general BLPs, we compare our universal model with these two predictors. In general, our predictor produce promising results with the mean AUC > 0.75. Moreover, three lineage-specific predictors all outperform corresponding universal ones, which empirically prove the effectiveness of the lineage-specific scheme. These results prove the good generalization capability of our method as well as the effectiveness of using lineage-specific strategy. To test if the improvement is statistically significant, we firstly use Shapiro-Wilk test [53] to check whether the data are normal. If it follows a normal distribution, we use student’s t-test [54]; otherwise, we use Wilcoxon signed-rank test [55]. A *p*-value less than 0.05 indicates the difference is statistically significant. This experiment demonstrates the improvement of our method is significant when compared with other predictors. Additionally, we are able to demonstrate our PredBLP significantly outperform the other predictors.

**Table 6:** Comparison of PredBLP with other methods on the independent testing dataset

Lineage	Predictor	Sensitivity	Specificity	Accuracy	MCC	AUC	*p*-value
General	BLProt	0.348 ± 0.022	0.903 ± 0.007	0.888 ± 0.007	0.132 ± 0.006	0.672 ± 0.010	0.002
	SCMBLP	0.471 ± 0.019	0.868 ± 0.008	0.858 ± 0.007	0.157 ± 0.004	N/A	0.002
	PredBLP-U	0.611 ± 0.013	0.921 ± 0.005	0.913 ± 0.004	0.294 ± 0.007	0.784 ± 0.007	N/A
Bacteria	BLProt	0.584 ± 0.020	0.769 ± 0.011	0.788 ± 0.010	0.166 ± 0.008	0.674 ± 0.008	0.002
	SCMBLP	0.569 ± 0.021	0.840 ± 0.013	0.831 ± 0.012	0.194 ± 0.005	N/A	0.002
	PredBLP-U	0.606 ± 0.015	0.909 ± 0.010	0.899 ± 0.009	0.299 ± 0.013	0.773 ± 0.009	0.002
	PredBLP-B	0.638 ± 0.017	0.927 ± 0.008	0.917 ± 0.007	0.352 ± 0.012	0.817	N/A
Eukaryota	BLProt	0.417 ± 0.037	0.966 ± 0.010	0.960 ± 0.010	0.212 ± 0.018	0.719 ± 0.016	0.002
	SCMBLP	0.667 ± 0.053	0.914 ± 0.014	0.912 ± 0.013	0.209 ± 0.009	N/A	0.002
	PredBLP-U	0.642 ± 0.038	0.954 ± 0.007	0.951 ± 0.006	0.279 ± 0.011	0.765 ± 0.007	0.004
	PredBLP-E	0.750 ± 0.037	0.946 ± 0.006	0.944 ± 0.005	0.301 ± 0.010	0.836 ± 0.006	N/A
Archaea	BLProt	0.583 ± 0.057	0.842 ± 0.016	0.838 ± 0.015	0.120 ± 0.010	0.666 ± 0.007	0.002
	SCMBLP	0.550 ± 0.061	0.883 ± 0.013	0.878 ± 0.012	0.154 ± 0.019	N/A	0.002
	PredBLP-U	0.775 ± 0.050	0.893 ± 0.014	0.891 ± 0.013	0.244 ± 0.012	0.751 ± 0.010	0.002
	PredBLP-A	0.750 ± 0.056	0.922 ± 0.012	0.920 ± 0.011	0.279 ± 0.012	0.789 ± 0.010	N/A

### Application to newly deposited BLPs in UniProt

The computational tools are often used to identify unknown proteins in real-life. Considering this, we collect BLPs that were deposited from August 2016 to February 2017 in UniProt. Next, we build four types of datasets, including general BLPs together with bacteria, eukaryota, and archaea BLPs. We random pick 80% BLPs as the testing dataset and repeat this procedure for 10 times as stated in section “Comparison with other predictors on independent testing datasets”. Here, we compare our webserver PredBLP with BLProt and SCMBLP. To achieve a fair comparison, we use the default parameters for these three predictors.

As listed in Table 7, for general BLPs, the proposed PredBLP-U correctly identify about 90% BLPs, which is 10% more than that for SCMBLP and BLProt. The *p*-value indicates the improvement is statistically significant. We see the similar results for bacteria BLPs and archaea BLPs. Especially, the lineage-special models all perform better results than that of the universal model. Both SCMBLP and PredBLP recognize more than 95% of archaea BLPs. However, although the lineage-specific model gives out higher results, the improvements are not statistically significant than that of universal model. The limited number of archaea BLPs could be the reason that account for this.

**Table 7:** Comparison of PredBLP with other methods on newly deposited BLPs

Lineage	Number of newly deposited BLPs	Predictor	Fraction of correctly identified BLPs	*p*-value
General	3741	BLProt	0.621 ± 0.013	0.002
		SCMBLP	0.792 ± 0.012	0.002
		PredBLP-U	0.889 ± 0.016	N/A
Bacteria	3614	BLProt	0.625 ± 0.022	0.002
		SCMBLP	0.795 ± 0.016	0.002
		PredBLP-U	0.887 ± 0.016	0.037
		PredBLP-B	0.912 ± 0.015	N/A
Eukaryota	106	BLProt	0.841 ± 0.041	0.002
		SCMBLP	0.908 ± 0.032	0.002
		PredBLP-U	0.651 ± 0.031	0.002
		PredBLP-E	0.983 ± 0.013	N/A
Archaea	21	BLProt	0.497 ± 0.046	0.002
		SCMBLP	0.954 ± 0.024	0.031
		PredBLP-U	0.980 ± 0.029	0.625
		PredBLP-A	0.993 ± 0.024	N/A

## Conclusion

In this study, we propose a novel predictor for the identification of BLPs by using sequence-derived features and lineage-specific scheme. Experiment on benchmark datasets proves the robustness and effectiveness of our method. We ascribe the good performance of the proposed method to three aspects. First, we collect the features which are capable to reflect the intrinsic properties of BLPs against non-BLPs. These features are also capable to distinguish various lineages of BLPs. Second, the effectiveness of the feature selection procedure. We successfully select the majority of the informative features as well as remove noisy features. Third, the introduction of lineage-specific strategy, which is proved to be more powerful than traditional universal approaches. Actually, the lineage-specific strategy is firstly introduced in this field. It is featured by characterizing the BLPs in a more specific way. The prediction performance on independent testing dataset and newly deposited BLPs in UniProt demonstrates that our method has a good generalization capability and is capable to exceed many state-of-art methods. Additionally, we empirically show that our predictor would be competitive when compared with currently public predictors.

## Additional file

Additional file 1:
**Table A1.** Physicochemical properties for twenty amino acids. **Table A2.** The relative amino acid composition of BLPs. **Table A3.** The relative dipeptide composition of general BLPs. **Table A4.** The relative dipeptide composition of bacteria BLPs. **Table A5.** The relative dipeptide composition of eukaryota BLPs. **Table A6.** The relative dipeptide composition of archaea BLPs. **Table A7.** The performance of different features and their combinations on three training sets using five-fold cross-validation. **Table A8.** The lists of optimum feature subsets in four training sets. **Figure A1.** An overview of the importance of the features in four training sets. **Figure A2.** Venn diagrams of the overlap between the discriminatory and selected useful features in the optimal subset for each type of features. (DOCX 1741 kb)

## Abbreviations

AAC: Amino acid composition
AUC: Area under receiver operating characteristic curve
DC: Dipeptide composition
DIG: Difference in information gain
IG: Information gain
MCC: Matthew’s correlation coefficients
PCP: Physicochemical properties
pKa: Protein kinase A
PredBLP: Prediction of bioluminescent proteins
PredBLP-A: Archaea-specific model of PredBLP
PredBLP-B: Bacteria-specific model of PredBLP
PredBLP-E: Eukaryota-specific model of PredBLP
PredBLP-U: Universal model of PredBLP
ROC: Receiver operating characteristic
