# Supplementary material for: Prediction of bioluminescent proteins by using sequence-derived features and lineage-specific scheme
Source: BMC Bioinformatics. 2017 Jun 5;18:294. doi: 10.1186/s12859-017-1709-6 (PMC5460367; doi:10.1186/s12859-017-1709-6)
Supplement: Additional file 1: — Table A1. Physicochemical properties for twenty amino acids. Table A2. The relative amino acid composition of BLPs. Table A3. The relative dipeptide composition of general BLPs. Table A4. The relative dipeptide composition of bacteria BLPs. Table A5. The relative dipeptide composition of eukaryota BLPs. Table A6. The relative dipeptide composition of archaea BLPs. Table A7. The performance of different features and their combinations on three training sets using five-fold cross-validation. Table A8. The lists of optimum feature subsets in four training sets. Figure A1. An overview of the importance of the features in four training sets. Figure A2. Venn diagrams of the overlap between the discriminatory and selected useful features in the optimal subset for each type of features. (DOCX 1741 kb) [file 12859_2017_1709_MOESM1_ESM.docx]

Additional File for

Prediction of bioluminescent proteins by using sequence-derived features and lineage-specific scheme

Jian Zhang^1, 2^, Haiting Chai^1^, Guifu Yang^1^, Zhiqiang Ma^1, *^

^1^School of Computer Science and Information Technology, Northeast Normal University, Changchun, Jilin Province, 130117, P.R. China.

^2^ School of Computer and Information Technology, Xinyang Normal University, Xinyang, Henan Province, 464000, P.R. China.

*Correspondence: zhiqiangma.nenu@gmail.com

The PDF file includes:

**Table A1** Physicochemical properties for twenty amino acids.

**Table A2** The relative amino acid composition of BLPs.

**Table A3** The relative dipeptide composition of general BLPs.

**Table A4** The relative dipeptide composition of bacteria BLPs.

**Table A5** The relative dipeptide composition of eukaryota BLPs.

**Table A6** The relative dipeptide composition of archaea BLPs.

**Table A7** The performance of different features and their combinations on three training sets using five-fold cross-validation.

**Table A8** The lists of optimum feature subsets in four training sets.

**Figure A1** An overview of the importance of the features in four training sets.

**Figure A2** Venn diagrams of the overlap between the discriminatory and selected useful features in the optimal subset for each type of features.

**Additional Table A1** Physicochemical properties for twenty amino acids.

|  | Hydrophobicity | Hydrophilicity | Polarity | Polarizability | Transfer free energy | Solvent contact area | Positively charge | Flexibility | Protein kinase A |
| --- | --- | --- | --- | --- | --- | --- | --- | --- | --- |
| A | -0.41 | -0.51 | 8.20 | 0.05 | 0.31 | 1.15 | 0.00 | 0.305 | 7.00 |
| R | -0.59 | 3.00 | 10.50 | 0.29 | -1.42 | 2.25 | 1.00 | 0.227 | 12.48 |
| D | -1.32 | 3.10 | 13.10 | 0.11 | -0.61 | 1.50 | 0.00 | 0.335 | 3.65 |
| C | 0.18 | -1.10 | 5.40 | 0.13 | 0.89 | 1.35 | 0.00 | 0.339 | 7.00 |
| Q | -0.91 | 0.20 | 10.50 | 0.18 | -0.71 | 1.83 | 0.00 | 0.306 | 7.00 |
| E | -1.23 | 3.20 | 12.40 | 0.15 | -0.71 | 1.90 | 0.00 | 0.282 | 3.22 |
| H | -0.65 | -0.51 | 10.50 | 0.24 | -0.12 | 1.95 | 1.00 | 0.215 | 6.00 |
| I | 1.26 | -1.70 | 5.30 | 0.19 | 0.71 | 1.75 | 0.00 | 0.278 | 7.00 |
| G | -0.67 | 0.00 | 9.00 | 0.00 | 0.32 | 0.75 | 0.00 | 0.352 | 7.00 |
| N | -0.92 | 0.22 | 11.60 | 0.13 | -0.48 | 1.60 | 0.00 | 0.322 | 8.18 |
| L | 1.22 | -1.80 | 4.91 | 0.19 | 0.51 | 1.70 | 0.00 | 0.262 | 7.00 |
| K | -0.67 | 3.00 | 11.30 | 0.22 | -1.80 | 2.00 | 1.00 | 0.391 | 10.53 |
| M | 1.04 | -1.31 | 5.70 | 0.22 | 0.41 | 1.85 | 0.00 | 0.28 | 7.00 |
| F | 1.93 | -2.40 | 5.30 | 0.28 | 0.49 | 2.10 | 0.00 | 0.195 | 7.00 |
| P | -0.49 | 0.00 | 8.10 | 0.13 | -0.31 | 1.45 | 0.00 | 0.346 | 7.00 |
| S | -0.55 | 0.30 | 9.20 | 0.06 | -0.13 | 1.16 | 0.00 | 0.326 | 7.00 |
| T | -0.28 | -0.40 | 8.60 | 0.11 | -0.20 | 1.42 | 0.00 | 0.251 | 7.00 |
| W | 0.51 | -3.40 | 5.40 | 0.41 | 0.31 | 2.58 | 0.00 | 0.291 | 7.00 |
| Y | 1.67 | -2.31 | 6.20 | 0.30 | -0.40 | 2.34 | 0.00 | 0.293 | 10.07 |
| V | 0.91 | -1.50 | 5.90 | 0.15 | 0.59 | 1.57 | 0.00 | 0.291 | 7.00 |

**Additional Table A2** The relative amino acid composition of BLPs.

| Amino acid types | General BLPs | Bacteria BLPs | Eukaryota BLPs | Archaea BLPs |
| --- | --- | --- | --- | --- |
| A | 1.81% | 2.09% | -0.78% | 1.89% |
| R | 1.56% | 2.05% | -0.96% | 0.80% |
| D | 0.00% | -0.12% | 0.35% | 1.49% |
| C | 0.05% | 0.07% | -0.26% | 0.25% |
| Q | -0.39% | -0.51% | -0.81% | 0.49% |
| E | -0.45% | -0.28% | -0.47% | -0.58% |
| H | 0.45% | 0.46% | 0.18% | 0.46% |
| I | -0.81% | -0.85% | 1.05% | -2.27% |
| G | 0.44% | 0.39% | 0.50% | 0.45% |
| N | -1.30% | -1.61% | 0.44% | -0.98% |
| L | 0.24% | 0.39% | -0.35% | -0.64% |
| K | -2.32% | -2.37% | -0.21% | -3.85% |
| M | -0.13% | -0.17% | 0.04% | -0.02% |
| F | 0.24% | 0.18% | 0.50% | 0.58% |
| P | 1.19% | 1.31% | -0.19% | 1.28% |
| S | -0.19% | -0.29% | -0.13% | 0.48% |
| T | -0.09% | -0.31% | 0.21% | 0.82% |
| W | 0.33% | 0.34% | -0.07% | 0.73% |
| Y | -0.41% | -0.52% | 0.36% | -0.35% |
| V | -0.22% | -0.25% | 0.60% | -1.03% |

*The colored blocks indicate discriminatory amino acid composition. They represent the absolute difference higher than 0.25%.*

**Additional Table A3** The relative dipeptide composition of general BLPs.

| V | 0.92 | 0.36 | -0.03 | 0.00 | -0.13 | -0.33 | -0.02 | -0.39 | 0.39 | -0.41 | 0.09 | -0.82 | 0.00 | -0.15 | 0.31 | -0.18 | -0.07 | 0.19 | -0.27 | -0.33 |
| --- | --- | --- | --- | --- | --- | --- | --- | --- | --- | --- | --- | --- | --- | --- | --- | --- | --- | --- | --- | --- |
| Y | 0.00 | 0.12 | -0.09 | -0.01 | -0.04 | -0.11 | 0.04 | -0.25 | -0.05 | -0.21 | -0.07 | -0.25 | -0.04 | -0.10 | 0.04 | -0.10 | -0.25 | 0.03 | -0.18 | -0.13 |
| W | 0.14 | 0.13 | 0.07 | 0.04 | -0.01 | 0.00 | 0.06 | 0.13 | 0.05 | 0.02 | 0.10 | -0.05 | 0.04 | 0.05 | 0.09 | 0.11 | 0.17 | 0.01 | -0.04 | 0.22 |
| T | 0.31 | 0.19 | -0.03 | -0.01 | -0.16 | 0.04 | 0.03 | -0.17 | -0.07 | -0.26 | 0.00 | -0.36 | 0.04 | -0.02 | 0.22 | 0.03 | 0.00 | 0.00 | -0.06 | -0.13 |
| S | 0.22 | 0.17 | -0.22 | -0.03 | -0.20 | -0.32 | 0.05 | -0.10 | 0.02 | -0.26 | -0.10 | -0.49 | 0.01 | 0.07 | 0.57 | 0.04 | -0.12 | 0.01 | -0.06 | -0.04 |
| P | 0.80 | 0.63 | 0.27 | 0.01 | 0.04 | 0.16 | 0.27 | 0.20 | 0.14 | -0.11 | 0.60 | -0.16 | 0.14 | 0.22 | 0.45 | 0.21 | 0.15 | 0.14 | 0.12 | 0.45 |
| F | 0.46 | 0.10 | 0.25 | 0.05 | -0.04 | 0.03 | 0.17 | -0.16 | 0.48 | -0.18 | -0.07 | -0.19 | -0.07 | 0.01 | 0.13 | 0.05 | -0.05 | 0.07 | -0.06 | -0.01 |
| M | -0.06 | 0.09 | -0.08 | 0.02 | -0.03 | -0.09 | -0.01 | -0.08 | -0.07 | -0.15 | 0.04 | -0.23 | -0.04 | 0.02 | 0.17 | -0.04 | 0.07 | 0.03 | -0.04 | -0.01 |
| K | -0.82 | -0.45 | -0.60 | -0.06 | -0.29 | -0.90 | -0.09 | -0.48 | -0.70 | -0.46 | -0.75 | -0.96 | -0.12 | -0.15 | -0.26 | -0.46 | -0.56 | -0.12 | -0.24 | -0.75 |
| L | 1.34 | 0.52 | 0.35 | 0.03 | -0.17 | -0.36 | 0.09 | -0.44 | 1.07 | -0.50 | -0.11 | -1.08 | -0.09 | -0.09 | 0.52 | 0.17 | -0.13 | 0.31 | -0.24 | -0.23 |
| N | -0.48 | -0.18 | -0.29 | -0.04 | -0.25 | -0.36 | -0.05 | -0.29 | -0.61 | -0.35 | -0.39 | -0.51 | -0.06 | -0.05 | -0.13 | -0.34 | -0.31 | -0.10 | -0.15 | -0.26 |
| G | 0.17 | 1.28 | -0.09 | -0.01 | -0.19 | -0.13 | 0.08 | -0.21 | 0.26 | -0.32 | 0.17 | -0.92 | -0.03 | 0.40 | 0.48 | -0.02 | 0.24 | 0.28 | 0.05 | 0.26 |
| I | 0.07 | 0.05 | -0.15 | 0.04 | -0.14 | -0.34 | -0.12 | -0.38 | 0.10 | -0.43 | -0.27 | -0.57 | -0.01 | -0.12 | 0.01 | -0.32 | -0.27 | 0.16 | -0.23 | -0.32 |
| H | 0.14 | 0.15 | 0.11 | 0.02 | -0.03 | 0.07 | 0.30 | 0.00 | 0.10 | -0.05 | 0.24 | -0.12 | 0.00 | 0.21 | 0.43 | 0.03 | 0.01 | 0.02 | 0.05 | 0.13 |
| E | 0.20 | 0.47 | -0.27 | 0.04 | 0.11 | -0.32 | 0.37 | -0.40 | -0.35 | -0.49 | 0.07 | -0.82 | -0.10 | 0.11 | 0.16 | -0.17 | -0.06 | -0.02 | -0.03 | -0.29 |
| Q | -0.03 | 0.16 | -0.15 | 0.02 | -0.18 | -0.18 | -0.01 | -0.05 | -0.25 | -0.17 | -0.04 | -0.27 | 0.00 | -0.03 | 0.03 | -0.11 | -0.06 | -0.02 | -0.14 | -0.04 |
| C | 0.11 | 0.05 | 0.02 | -0.03 | 0.01 | -0.01 | -0.03 | 0.00 | 0.00 | -0.04 | 0.02 | -0.06 | 0.01 | 0.03 | -0.01 | 0.10 | 0.00 | 0.05 | -0.03 | 0.00 |
| D | 0.43 | 0.54 | -0.06 | 0.00 | 0.12 | 0.15 | 0.31 | -0.33 | 0.07 | -0.31 | -0.06 | -0.53 | -0.14 | -0.03 | 0.34 | -0.18 | -0.03 | -0.03 | -0.20 | -0.05 |
| R | 1.01 | 0.80 | 0.41 | 0.03 | -0.03 | 0.23 | 0.25 | 0.31 | 0.30 | -0.15 | 1.01 | -0.36 | 0.11 | 0.43 | 0.63 | 0.27 | 0.47 | 0.07 | 0.09 | 0.33 |
| A | 2.31 | 1.09 | 0.57 | 0.07 | 0.05 | 0.94 | 0.12 | -0.20 | 0.86 | -0.38 | 0.46 | -0.58 | 0.06 | 0.14 | 0.56 | 0.14 | 0.43 | 0.25 | 0.00 | 0.31 |
|  | A | R | D | C | Q | E | H | I | G | N | L | K | M | F | P | S | T | W | Y | V |

*The colored blocks indicate discriminatory dipeptide composition. In detail, red blocks show the difference higher than 1.00, light red ones indicate the difference betwen 0.50 and 1.00; green and light green ones stand for the difference lower than -1.00 and range from -1.00 to -0.50, respectively.*

**Additional Table A4** The relative dipeptide composition of bacteria BLPs.

| V | 1.02 | 0.45 | -0.04 | -0.01 | -0.15 | -0.28 | -0.01 | -0.43 | 0.42 | -0.55 | 0.13 | -0.84 | -0.04 | -0.18 | 0.34 | -0.27 | -0.12 | 0.20 | -0.28 | -0.35 |
| --- | --- | --- | --- | --- | --- | --- | --- | --- | --- | --- | --- | --- | --- | --- | --- | --- | --- | --- | --- | --- |
| Y | -0.02 | 0.16 | -0.13 | -0.02 | -0.08 | -0.11 | 0.02 | -0.27 | -0.12 | -0.26 | -0.06 | -0.27 | -0.06 | -0.12 | 0.03 | -0.13 | -0.31 | 0.04 | -0.24 | -0.16 |
| W | 0.16 | 0.16 | 0.07 | 0.04 | -0.02 | 0.01 | 0.06 | 0.13 | 0.05 | 0.01 | 0.08 | -0.05 | 0.03 | 0.04 | 0.08 | 0.13 | 0.17 | 0.02 | -0.04 | 0.21 |
| T | 0.33 | 0.21 | -0.09 | -0.01 | -0.21 | -0.03 | 0.02 | -0.23 | -0.22 | -0.35 | -0.04 | -0.40 | 0.01 | -0.05 | 0.21 | -0.01 | -0.09 | 0.00 | -0.11 | -0.19 |
| S | 0.24 | 0.22 | -0.33 | -0.02 | -0.21 | -0.36 | 0.01 | -0.14 | -0.04 | -0.33 | -0.08 | -0.51 | 0.00 | 0.06 | 0.63 | 0.04 | -0.20 | -0.03 | -0.07 | -0.12 |
| P | 0.92 | 0.81 | 0.26 | 0.02 | 0.05 | 0.17 | 0.30 | 0.21 | 0.21 | -0.15 | 0.64 | -0.19 | 0.12 | 0.22 | 0.57 | 0.23 | 0.08 | 0.15 | 0.14 | 0.48 |
| F | 0.53 | 0.14 | 0.19 | 0.04 | -0.05 | 0.04 | 0.14 | -0.19 | 0.55 | -0.23 | -0.01 | -0.23 | -0.09 | -0.01 | 0.13 | -0.03 | -0.10 | 0.07 | -0.10 | -0.04 |
| M | -0.06 | 0.11 | -0.08 | 0.02 | -0.05 | -0.08 | -0.01 | -0.07 | -0.11 | -0.19 | 0.04 | -0.25 | -0.06 | -0.01 | 0.17 | -0.04 | 0.02 | 0.03 | -0.06 | -0.01 |
| K | -0.89 | -0.39 | -0.66 | -0.06 | -0.34 | -0.82 | -0.13 | -0.45 | -0.78 | -0.50 | -0.73 | -0.83 | -0.14 | -0.15 | -0.28 | -0.51 | -0.63 | -0.11 | -0.24 | -0.76 |
| L | 1.62 | 0.73 | 0.37 | 0.05 | -0.17 | -0.29 | 0.09 | -0.45 | 1.27 | -0.59 | -0.06 | -1.13 | -0.09 | -0.14 | 0.48 | 0.20 | -0.21 | 0.35 | -0.25 | -0.22 |
| N | -0.55 | -0.17 | -0.37 | -0.05 | -0.31 | -0.39 | -0.08 | -0.37 | -0.79 | -0.46 | -0.48 | -0.55 | -0.08 | -0.11 | -0.20 | -0.44 | -0.41 | -0.12 | -0.21 | -0.30 |
| G | 0.20 | 1.52 | -0.21 | 0.00 | -0.24 | -0.15 | 0.08 | -0.21 | 0.27 | -0.47 | 0.17 | -1.02 | -0.07 | 0.42 | 0.57 | 0.03 | 0.25 | 0.28 | -0.07 | 0.19 |
| I | 0.06 | 0.14 | -0.21 | 0.05 | -0.17 | -0.29 | -0.13 | -0.33 | 0.09 | -0.52 | -0.25 | -0.57 | 0.00 | -0.14 | -0.01 | -0.41 | -0.34 | 0.18 | -0.23 | -0.33 |
| H | 0.13 | 0.20 | 0.12 | 0.03 | -0.05 | 0.07 | 0.33 | -0.02 | 0.10 | -0.07 | 0.27 | -0.14 | -0.01 | 0.22 | 0.45 | 0.03 | 0.00 | 0.03 | 0.02 | 0.11 |
| E | 0.24 | 0.63 | -0.19 | 0.03 | 0.08 | -0.12 | 0.43 | -0.31 | -0.43 | -0.54 | 0.20 | -0.80 | -0.15 | 0.15 | 0.21 | -0.17 | -0.12 | -0.01 | -0.04 | -0.19 |
| Q | -0.08 | 0.22 | -0.16 | 0.03 | -0.22 | -0.18 | -0.01 | -0.09 | -0.32 | -0.23 | -0.04 | -0.31 | -0.02 | -0.06 | 0.04 | -0.16 | -0.10 | -0.03 | -0.19 | -0.09 |
| C | 0.13 | 0.11 | 0.02 | 0.00 | -0.02 | -0.01 | -0.05 | -0.02 | 0.02 | -0.03 | 0.04 | -0.03 | 0.00 | 0.02 | 0.01 | 0.10 | -0.01 | 0.05 | -0.03 | -0.01 |
| D | 0.44 | 0.60 | -0.18 | -0.02 | 0.10 | 0.19 | 0.30 | -0.37 | -0.07 | -0.39 | -0.01 | -0.57 | -0.15 | -0.05 | 0.37 | -0.25 | -0.09 | -0.04 | -0.22 | -0.07 |
| R | 1.20 | 1.06 | 0.48 | 0.05 | -0.03 | 0.35 | 0.31 | 0.44 | 0.48 | -0.15 | 1.25 | -0.26 | 0.11 | 0.51 | 0.77 | 0.38 | 0.53 | 0.08 | 0.12 | 0.47 |
| A | 2.76 | 1.30 | 0.65 | 0.09 | 0.03 | 1.16 | 0.16 | -0.26 | 0.99 | -0.46 | 0.53 | -0.56 | 0.04 | 0.12 | 0.67 | 0.08 | 0.44 | 0.23 | 0.00 | 0.36 |
|  | A | R | D | C | Q | E | H | I | G | N | L | K | M | F | P | S | T | W | Y | V |

*The colored blocks indicate discriminatory dipeptide composition. In detail, red blocks show the difference higher than 1.00, light red ones indicate the difference betwen 0.50 and 1.00; green and light green ones stand for the difference lower than -1.00 and range from -1.00 to -0.50, respectively.*

**Additional Table A5** The relative dipeptide composition of eukaryota BLPs.

| V | 0.24 | 0.04 | 0.54 | -0.01 | -0.37 | 0.30 | 0.13 | 0.51 | 0.36 | 0.46 | 0.10 | 0.20 | 0.27 | 0.10 | -0.07 | 0.04 | 0.19 | 0.01 | -0.11 | 0.39 |
| --- | --- | --- | --- | --- | --- | --- | --- | --- | --- | --- | --- | --- | --- | --- | --- | --- | --- | --- | --- | --- |
| Y | 0.20 | 0.00 | 0.10 | -0.01 | -0.09 | 0.11 | 0.13 | 0.21 | 0.60 | 0.10 | -0.06 | 0.10 | 0.04 | 0.00 | 0.10 | -0.10 | 0.09 | -0.03 | 0.13 | 0.47 |
| W | -0.16 | 0.01 | -0.09 | 0.00 | -0.01 | -0.10 | -0.09 | 0.11 | -0.06 | 0.04 | 0.01 | 0.01 | 0.07 | -0.01 | -0.03 | -0.16 | 0.04 | -0.01 | -0.07 | 0.10 |
| T | -0.24 | -0.04 | -0.06 | -0.11 | -0.13 | 0.23 | 0.14 | 0.19 | 0.86 | 0.23 | -0.29 | -0.16 | 0.30 | 0.07 | -0.17 | 0.11 | 0.23 | -0.01 | -0.01 | 0.03 |
| S | -0.57 | -0.34 | 0.01 | -0.17 | -0.74 | -0.09 | 0.21 | 0.31 | 0.23 | 0.00 | -0.30 | -0.14 | -0.04 | 0.14 | 0.00 | 0.23 | 0.11 | 0.13 | 0.01 | 0.26 |
| P | -0.69 | -0.53 | 0.09 | -0.10 | -0.49 | 0.27 | -0.04 | 0.10 | -0.57 | 0.23 | 0.64 | 0.44 | 0.36 | 0.19 | -0.89 | -0.17 | 0.17 | -0.17 | -0.03 | 0.06 |
| F | 0.11 | -0.21 | 0.66 | 0.01 | -0.10 | -0.16 | 0.41 | 0.23 | 0.09 | 0.16 | -0.51 | 0.60 | 0.04 | 0.17 | 0.20 | 0.41 | 0.01 | 0.06 | 0.30 | 0.33 |
| M | -0.49 | 0.04 | -0.26 | 0.00 | -0.01 | -0.11 | 0.04 | 0.10 | 0.17 | -0.01 | 0.10 | -0.04 | 0.01 | 0.24 | 0.07 | -0.19 | 0.40 | -0.04 | 0.09 | 0.13 |
| K | -0.26 | -0.64 | 0.23 | 0.06 | -0.41 | -0.39 | 0.29 | 0.76 | 0.51 | 0.03 | -0.17 | -1.20 | 0.17 | -0.07 | -0.19 | 0.03 | -0.23 | -0.17 | 0.29 | 0.26 |
| L | -0.53 | -0.33 | 0.37 | -0.40 | -0.76 | -0.29 | 0.06 | 0.44 | -0.50 | 0.10 | -0.73 | -0.16 | -0.13 | 0.23 | 0.74 | 0.09 | 0.01 | 0.07 | -0.11 | -0.27 |
| N | -0.01 | -0.31 | 0.24 | -0.06 | -0.14 | -0.01 | -0.06 | 0.43 | 0.56 | 0.50 | 0.34 | -0.04 | 0.14 | 0.49 | 0.20 | 0.06 | 0.19 | -0.21 | 0.26 | -0.01 |
| G | -0.54 | -0.41 | 0.66 | -0.10 | -0.21 | 0.17 | 0.09 | 0.29 | 0.59 | 0.41 | 0.13 | 0.14 | 0.34 | 0.39 | -0.20 | -0.53 | -0.13 | 0.13 | 0.96 | 0.60 |
| I | 0.49 | -0.10 | 0.69 | 0.10 | -0.29 | -0.03 | 0.07 | 0.29 | 0.89 | 0.56 | 0.50 | 0.43 | 0.17 | 0.14 | 0.29 | 0.71 | 0.33 | -0.06 | 0.10 | 0.74 |
| H | 0.23 | -0.17 | -0.10 | -0.13 | 0.10 | -0.04 | 0.13 | 0.34 | 0.00 | -0.10 | -0.03 | 0.07 | 0.04 | 0.00 | 0.43 | -0.11 | -0.04 | -0.04 | 0.21 | 0.29 |
| E | -0.03 | -0.36 | -1.14 | 0.19 | -0.16 | -1.16 | -0.10 | 0.31 | 0.23 | -0.03 | 0.11 | -0.16 | 0.26 | 0.16 | -0.24 | -0.53 | 0.24 | -0.11 | 0.11 | -0.29 |
| Q | -0.33 | -0.47 | -0.40 | -0.17 | -0.53 | -0.63 | -0.16 | 0.07 | -0.33 | -0.07 | -0.70 | -0.17 | 0.04 | 0.01 | -0.26 | -0.14 | -0.27 | -0.06 | -0.07 | -0.03 |
| C | -0.16 | -0.46 | -0.04 | -0.26 | 0.13 | -0.19 | 0.09 | 0.13 | -0.33 | -0.20 | -0.14 | -0.30 | 0.03 | 0.20 | -0.19 | 0.10 | 0.04 | 0.04 | -0.03 | 0.07 |
| D | -0.04 | 0.01 | 0.53 | 0.00 | 0.11 | 0.01 | 0.13 | 0.40 | 1.10 | 0.13 | -0.33 | -0.09 | -0.17 | 0.14 | 0.01 | -0.33 | 0.07 | -0.01 | -0.13 | 0.50 |
| R | -0.37 | -1.03 | 0.17 | -0.16 | -0.26 | -0.10 | -0.30 | 0.23 | -1.01 | 0.00 | -0.63 | -0.53 | -0.03 | -0.19 | -0.57 | -0.56 | -0.10 | 0.04 | -0.04 | -0.09 |
| A | -1.43 | -0.33 | -0.26 | -0.21 | -0.36 | -0.66 | -0.20 | 0.47 | -0.67 | -0.10 | -0.24 | -0.36 | 0.04 | 0.34 | -0.46 | 0.14 | -0.26 | 0.01 | 0.17 | -0.19 |
|  | A | R | D | C | Q | E | H | I | G | N | L | K | M | F | P | S | T | W | Y | V |

*The colored blocks indicate discriminatory dipeptide composition. In detail, red blocks show the difference higher than 1.00, light red ones indicate the difference betwen 0.50 and 1.00; green and light green ones stand for the difference lower than -1.00 and range from -1.00 to -0.50, respectively.*

**Additional Table A6** The relative dipeptide composition of archaea BLPs.

| V | 0.81 | 0.02 | -0.35 | 0.08 | -0.05 | -0.86 | -0.10 | -0.84 | 0.14 | -0.37 | -0.21 | -1.56 | 0.03 | -0.02 | 0.34 | 0.28 | 0.03 | 0.17 | -0.38 | -1.23 |
| --- | --- | --- | --- | --- | --- | --- | --- | --- | --- | --- | --- | --- | --- | --- | --- | --- | --- | --- | --- | --- |
| Y | 0.01 | 0.02 | 0.28 | -0.02 | -0.08 | -0.06 | 0.18 | -0.46 | 0.01 | -0.27 | -0.20 | -0.47 | 0.05 | 0.10 | -0.09 | 0.19 | -0.06 | 0.06 | -0.04 | -0.56 |
| W | 0.14 | 0.10 | 0.18 | 0.04 | 0.07 | 0.03 | 0.20 | 0.23 | 0.23 | 0.22 | 0.21 | -0.04 | 0.13 | 0.08 | 0.22 | 0.10 | 0.28 | -0.01 | 0.00 | 0.56 |
| T | 0.25 | 0.07 | 0.94 | 0.22 | -0.11 | 0.39 | -0.10 | 0.17 | 0.16 | -0.20 | 0.46 | -0.51 | 0.05 | 0.21 | 0.52 | -0.03 | 0.37 | -0.02 | 0.19 | 0.24 |
| S | 0.26 | 0.06 | 0.66 | 0.11 | -0.03 | -0.06 | 0.12 | -0.14 | 0.24 | -0.04 | -0.15 | -0.71 | 0.11 | 0.35 | 0.64 | 0.12 | 0.11 | 0.17 | -0.07 | 0.23 |
| P | 0.85 | 0.47 | 0.84 | -0.02 | 0.37 | 0.28 | 0.38 | 0.23 | -0.38 | -0.12 | 0.26 | -0.37 | 0.17 | 0.17 | 0.39 | 0.47 | 0.49 | 0.34 | -0.05 | 0.42 |
| F | 0.42 | 0.17 | 0.77 | 0.04 | -0.04 | 0.58 | 0.19 | -0.09 | 0.28 | 0.02 | -0.21 | -0.41 | -0.04 | -0.02 | 0.14 | 0.51 | 0.01 | 0.09 | -0.12 | -0.01 |
| M | 0.28 | -0.09 | 0.11 | -0.02 | 0.00 | -0.21 | -0.10 | -0.14 | -0.04 | 0.00 | 0.08 | -0.20 | 0.01 | -0.06 | 0.27 | 0.10 | 0.13 | 0.05 | -0.07 | -0.14 |
| K | -0.96 | -0.79 | -1.03 | -0.15 | -0.07 | -1.86 | -0.06 | -1.51 | -1.24 | -0.74 | -1.43 | -1.90 | -0.01 | -0.21 | -0.30 | -0.53 | -0.53 | -0.14 | -0.43 | -1.53 |
| L | 0.50 | -0.32 | 0.27 | 0.07 | 0.12 | -0.76 | 0.09 | -1.27 | 1.40 | -0.55 | -0.38 | -1.56 | -0.10 | -0.04 | 0.46 | 0.01 | -0.01 | 0.33 | -0.24 | -0.55 |
| N | -0.49 | -0.33 | -0.15 | 0.02 | -0.10 | -0.56 | 0.16 | -0.41 | -0.11 | -0.26 | -0.46 | -0.72 | -0.08 | -0.10 | -0.07 | -0.12 | -0.11 | -0.04 | 0.05 | -0.12 |
| G | 0.32 | 0.96 | 0.68 | -0.01 | -0.05 | 0.18 | -0.14 | -0.91 | -0.01 | -0.08 | 0.13 | -1.27 | -0.05 | 0.49 | 0.24 | -0.32 | 0.58 | 0.61 | -0.02 | 0.52 |
| I | 0.14 | -0.40 | -0.13 | 0.04 | 0.09 | -0.81 | -0.32 | -1.18 | -0.35 | -0.64 | -1.18 | -1.58 | -0.19 | -0.11 | -0.05 | -0.32 | -0.53 | 0.13 | -0.48 | -1.13 |
| H | 0.06 | -0.01 | 0.19 | 0.11 | 0.06 | 0.09 | 0.12 | -0.25 | 0.07 | 0.04 | 0.34 | -0.17 | -0.06 | 0.39 | 0.50 | 0.10 | 0.08 | 0.00 | 0.05 | 0.13 |
| E | 0.51 | 0.64 | 0.15 | 0.12 | 0.83 | -1.03 | 0.19 | -1.35 | 0.11 | -0.57 | -0.79 | -1.36 | 0.05 | -0.08 | 0.45 | 0.41 | 0.41 | -0.03 | 0.23 | -1.15 |
| Q | 0.30 | 0.11 | 0.02 | 0.05 | 0.12 | -0.07 | 0.06 | 0.15 | -0.17 | 0.08 | 0.51 | -0.08 | 0.01 | 0.22 | 0.00 | 0.10 | 0.23 | 0.17 | 0.10 | 0.04 |
| C | 0.23 | 0.03 | 0.03 | -0.01 | 0.21 | 0.17 | -0.03 | 0.12 | -0.14 | -0.04 | 0.08 | -0.02 | 0.01 | 0.04 | -0.07 | 0.05 | 0.06 | 0.12 | 0.05 | 0.09 |
| D | 0.94 | 1.05 | 0.88 | 0.21 | 0.30 | 0.81 | 0.86 | -0.68 | 0.90 | -0.02 | -0.21 | -0.55 | -0.21 | -0.10 | 0.62 | 0.58 | 0.07 | 0.00 | 0.02 | 0.38 |
| R | 0.96 | 0.86 | 0.42 | 0.00 | 0.03 | 0.43 | 0.18 | -0.59 | -0.34 | -0.32 | 0.60 | -0.84 | 0.29 | 0.56 | 0.54 | -0.16 | 0.71 | 0.15 | -0.02 | -0.43 |
| A | 2.03 | 0.58 | 1.17 | 0.11 | 0.26 | 0.98 | -0.05 | -0.19 | 1.03 | -0.11 | -0.05 | -1.14 | 0.03 | 0.43 | 0.37 | 0.41 | 0.92 | 0.80 | -0.18 | 0.12 |
|  | A | R | D | C | Q | E | H | I | G | N | L | K | M | F | P | S | T | W | Y | V |

*The colored blocks indicate discriminatory dipeptide composition. In detail, red blocks show the difference higher than 1.00, light red ones indicate the difference betwen 0.50 and 1.00; green and light green ones stand for the difference lower than -1.00 and range from -1.00 to -0.50, respectively.*

**Additional Table A7** The performance of different features and their combinations on three training sets using five-fold cross-validation.

| Lineage | Bacteria | | | | | Eukaryota | | | | | Archaea | | | | |
| --- | --- | --- | --- | --- | --- | --- | --- | --- | --- | --- | --- | --- | --- | --- | --- |
| Feature | Sensitivity | Specificity | Accuracy | MCC | AUC | Sensitivity | Specificity | Accuracy | MCC | AUC | Sensitivity | Specificity | Accuracy | MCC | AUC |
| AAC | 0.701  ±0.015 | 0.901  ±0.017 | 0.801  ±0.007 | 0.611  ±0.013 | 0.834  ±0.010 | 0.450  ±0.041 | 0.750  ±0.053 | 0.600  ±0.033 | 0.211  ±0.072 | 0.571  ±0.014 | 0.600  ±0.050 | 0.850  ±0.094 | 0.725  ±0.050 | 0.470  ±0.114 | 0.816  ±0.017 |
| DC | 0.766  ±0.019 | 0.918  ±0.013 | 0.842  ±0.009 | 0.700  ±0.008 | 0.917  ±0.006 | 0.483  ±0.062 | 0.867  ±0.085 | 0.675  ±0.017 | 0.389  ±0.064 | 0.726  ±0.021 | 0.725  ±0.094 | 0.775  ±0.050 | 0.750  ±0.040 | 0.505  ±0.080 | 0.830  ±0.020 |
| MTF | 0.575  ±0.012 | 0.938  ±0.016 | 0.756  ±0.007 | 0.556  ±0.018 | 0.769  ±0.006 | 0.500  ±0.053 | 0.850  ±0.097 | 0.675  ±0.041 | 0.384  ±0.106 | 0.698  ±0.015 | 0.550  ±0.061 | 0.875  ±0.079 | 0.713  ±0.031 | 0.456  ±0.077 | 0.817  ±0.019 |
| PCP | 0.697  ±0.009 | 0.751  ±0.014 | 0.724  ±0.004 | 0.452  ±0.014 | 0.758  ±0.011 | 0.417  ±0.053 | 0.783  ±0.041 | 0.600  ±0.020 | 0.216  ±0.042 | 0.573  ±0.018 | 0.325  ±0.061 | 0.875  ±0.079 | 0.600  ±0.031 | 0.250  ±0.090 | 0.593  ±0.024 |
| AAC+DC | 0.766  ±0.015 | 0.925  ±0.014 | 0.845  ±0.005 | 0.706  ±0.015 | 0.896  ±0.011 | 0.650  ±0.097 | 0.750  ±0.053 | 0.700  ±0.049 | 0.405  ±0.094 | 0.729  ±0.022 | 0.725  ±0.094 | 0.775  ±0.050 | 0.750  ±0.056 | 0.503  ±0.113 | 0.835  ±0.024 |
| AAC+MTF | 0.766  ±0.012 | 0.875  ±0.019 | 0.820  ±0.007 | 0.644  ±0.010 | 0.853  ±0.007 | 0.517  ±0.062 | 0.833  ±0.075 | 0.675  ±0.031 | 0.374  ±0.071 | 0.702  ±0.014 | 0.600  ±0.094 | 0.875  ±0.079 | 0.738  ±0.025 | 0.503  ±0.056 | 0.829  ±0.017 |
| AAC+PCP | 0.651  ±0.019 | 0.935  ±0.011 | 0.793  ±0.009 | 0.612  ±0.010 | 0.834  ±0.005 | 0.417  ±0.053 | 0.817  ±0.062 | 0.617  ±0.055 | 0.255  ±0.122 | 0.603  ±0.020 | 0.600  ±0.050 | 0.875  ±0.079 | 0.738  ±0.047 | 0.498  ±0.105 | 0.824  ±0.024 |
| DC+MTF | 0.810  ±0.016 | 0.893  ±0.017 | 0.851  ±0.004 | 0.704  ±0.014 | 0.900  ±0.006 | 0.633  ±0.085 | 0.767  ±0.062 | 0.700  ±0.031 | 0.407  ±0.060 | 0.740  ±0.015 | 0.550  ±0.061 | 0.925  ±0.061 | 0.738  ±0.047 | 0.515  ±0.104 | 0.834  ±0.017 |
| DC+PCP | 0.766  ±0.012 | 0.928  ±0.009 | 0.847  ±0.005 | 0.703  ±0.011 | 0.897  ±0.010 | 0.650  ±0.062 | 0.750  ±0.053 | 0.700  ±0.017 | 0.405  ±0.034 | 0.731  ±0.017 | 0.500  ±0.112 | 0.950  ±0.061 | 0.725  ±0.075 | 0.504  ±0.151 | 0.827  ±0.027 |
| MTF+PCP | 0.743  ±0.019 | 0.883  ±0.014 | 0.813  ±0.007 | 0.628  ±0.014 | 0.839  ±0.006 | 0.467  ±0.067 | 0.883  ±0.041 | 0.675  ±0.031 | 0.387  ±0.062 | 0.720  ±0.015 | 0.575  ±0.061 | 0.875  ±0.079 | 0.725  ±0.031 | 0.479  ±0.075 | 0.823  ±0.016 |
| AAC+DC+MTF | 0.743  ±0.012 | 0.957  ±0.016 | 0.850  ±0.009 | 0.716  ±0.013 | 0.907  ±0.007 | 0.617  ±0.041 | 0.817  ±0.062 | 0.717  ±0.017 | 0.445  ±0.043 | 0.744  ±0.015 | 0.625  ±0.079 | 0.875  ±0.112 | 0.750  ±0.040 | 0.529  ±0.097 | 0.838  ±0.022 |
| AAC+DC+PCP | 0.766  ±0.021 | 0.934  ±0.013 | 0.850  ±0.005 | 0.707  ±0.010 | 0.899  ±0.005 | 0.583  ±0.053 | 0.817  ±0.062 | 0.700  ±0.041 | 0.413  ±0.088 | 0.742  ±0.016 | 0.725  ±0.094 | 0.800  ±0.061 | 0.763  ±0.073 | 0.527  ±0.145 | 0.843  ±0.026 |
| AAC+MTF+PCP | 0.758  ±0.011 | 0.940  ±0.012 | 0.849  ±0.009 | 0.713  ±0.015 | 0.880  ±0.004 | 0.517  ±0.062 | 0.850  ±0.033 | 0.683  ±0.033 | 0.390  ±0.067 | 0.728  ±0.012 | 0.550  ±0.100 | 0.925  ±0.061 | 0.738  ±0.047 | 0.518  ±0.088 | 0.839  ±0.016 |
| DC+MTF+PCP | 0.738  ±0.009 | 0.957  ±0.019 | 0.847  ±0.007 | 0.714  ±0.010 | 0.907  ±0.006 | 0.583  ±0.075 | 0.817  ±0.062 | 0.700  ±0.017 | 0.416  ±0.034 | 0.740  ±0.015 | 0.575  ±0.061 | 0.925  ±0.061 | 0.750  ±0.040 | 0.538  ±0.088 | 0.850  ±0.019 |
| AAC+DC+MTF+PCP | 0.732  ±0.016 | 0.972  ±0.019 | 0.852  ±0.006 | 0.725  ±0.014 | 0.909  ±0.009 | 0.717  ±0.067 | 0.767  ±0.062 | 0.742  ±0.031 | 0.487  ±0.063 | 0.769  ±0.018 | 0.650  ±0.094 | 0.900  0.094 | 0.775  ±0.031 | 0.580  ±0.066 | 0.857  ±0.022 |

*AAC, DC, MTF and PCP represent the features of amino acid composition, dipeptide composition, sequence motifs and physicochemical properties respectively.*

**Supplementary Table A8** The lists of optimum feature subsets in four training sets.

| Lineage | Features |
| --- | --- |
| General | AAC.[A], AAC.[R], AAC.[E], AAC.[I], AAC.[G], AAC.[L], AAC.[K], DC.[AA], DC.[AR], DC.[AD], DC.[AQ], DC.[AE], DC.[AI], DC.[AG], DC.[AN], DC.[AL], DC.[AK], DC.[AF], DC.[AP], DC.[AS], DC.[AT], DC.[AV], DC.[RA], DC.[RR], DC.[RD], DC.[RE], DC.[RI], DC.[RG], DC.[RL], DC.[RK], DC.[RF], DC.[RP], DC.[RS], DC.[RT], DC.[RV], DC.[DA], DC.[DR], DC.[DD], DC.[DE], DC.[DI], DC.[DG], DC.[DL], DC.[DK], DC.[DF], DC.[DP], DC.[DS], DC.[DT], DC.[DV], DC.[QA], DC.[QE], DC.[QL], DC.[EA], DC.[ER], DC.[ED], DC.[EQ], DC.[EE], DC.[EI], DC.[EG], DC.[EN], DC.[EL], DC.[EK], DC.[EF], DC.[EP], DC.[ES], DC.[ET], DC.[EV], DC.[HP], DC.[IA], DC.[IR], DC.[ID], DC.[IE], DC.[II], DC.[IG], DC.[IN], DC.[IL], DC.[IK], DC.[IP], DC.[IS], DC.[IT], DC.[IV], DC.[GA], DC.[GR], DC.[GD], DC.[GE], DC.[GI], DC.[GG], DC.[GN], DC.[GL], DC.[GK], DC.[GF], DC.[GP], DC.[GS], DC.[GT], DC.[GW], DC.[GY], DC.[GV], DC.[NA], DC.[NI], DC.[NG], DC.[NL], DC.[NK], DC.[NV], DC.[LA], DC.[LR], DC.[LD], DC.[LQ], DC.[LE], DC.[LI], DC.[LG], DC.[LN], DC.[LL], DC.[LK], DC.[LF], DC.[LP], DC.[LS], DC.[LT], DC.[LY], DC.[LV], DC.[KA], DC.[KR], DC.[KD], DC.[KQ], DC.[KE], DC.[KI], DC.[KG], DC.[KN], DC.[KL], DC.[KK], DC.[KP], DC.[KS], DC.[KT], DC.[KV], DC.[MA], DC.[FA], DC.[FD], DC.[FE], DC.[FG], DC.[FL], DC.[FS], DC.[FV], DC.[PA], DC.[PR], DC.[PD], DC.[PE], DC.[PI], DC.[PG], DC.[PL], DC.[PP], DC.[PS], DC.[PT], DC.[PV], DC.[SA], DC.[SR], DC.[SD], DC.[SE], DC.[SI], DC.[SG], DC.[SN], DC.[SL], DC.[SK], DC.[SF], DC.[SP], DC.[SS], DC.[ST], DC.[SV], DC.[TA], DC.[TR], DC.[TD], DC.[TE], DC.[TI], DC.[TG], DC.[TL], DC.[TK], DC.[TP], DC.[TS], DC.[TT], DC.[TV], DC.[YA], DC.[YG], DC.[YL], DC.[VA], DC.[VR], DC.[VD], DC.[VE], DC.[VI], DC.[VG], DC.[VN], DC.[VL], DC.[VK], DC.[VP], DC.[VS], DC.[VT], DC.[VV], MTF.[EH-H], MTF.[L-S-GR], MTF.[L-G-GR], PCP.[Transfer free energy], PCP.[Positively charge], PCP.[Flexibility]. (Total number: 199) |
| Bacteria | AAC.[A], AAC.[R], AAC.[G], AAC.[L], AAC.[K], DC.[AA], DC.[AR], DC.[AD], DC.[AQ], DC.[AE], DC.[AI], DC.[AG], DC.[AL], DC.[AK], DC.[AF], DC.[AP], DC.[AS], DC.[AT], DC.[AV], DC.[RA], DC.[RR], DC.[RD], DC.[RE], DC.[RI], DC.[RG], DC.[RL], DC.[RK], DC.[RF], DC.[RP], DC.[RS], DC.[RT], DC.[RV], DC.[DA], DC.[DR], DC.[DD], DC.[DE], DC.[DI], DC.[DG], DC.[DL], DC.[DK], DC.[DP], DC.[DS], DC.[DV], DC.[QA], DC.[QR], DC.[QG], DC.[QL], DC.[EA], DC.[ER], DC.[ED], DC.[EQ], DC.[EE], DC.[EI], DC.[EG], DC.[EL], DC.[EK], DC.[ES], DC.[ET], DC.[EV], DC.[IA], DC.[IR], DC.[ID], DC.[IE], DC.[II], DC.[IG], DC.[IL], DC.[IK], DC.[IS], DC.[IT], DC.[IV], DC.[GA], DC.[GR], DC.[GD], DC.[GE], DC.[GI], DC.[GG], DC.[GN], DC.[GL], DC.[GK], DC.[GF], DC.[GP], DC.[GS], DC.[GT], DC.[GY], DC.[GV], DC.[NA], DC.[NI], DC.[NG], DC.[NL], DC.[LA], DC.[LR], DC.[LD], DC.[LQ], DC.[LE], DC.[LI], DC.[LG], DC.[LN], DC.[LL], DC.[LK], DC.[LF], DC.[LP], DC.[LS], DC.[LT], DC.[LV], DC.[KA], DC.[KD], DC.[KQ], DC.[KE], DC.[KI], DC.[KG], DC.[KN], DC.[KL], DC.[KK], DC.[KS], DC.[KT], DC.[KV], DC.[FA], DC.[FD], DC.[FE], DC.[FG], DC.[FL], DC.[FV], DC.[PA], DC.[PR], DC.[PD], DC.[PE], DC.[PG], DC.[PL], DC.[PP], DC.[PV], DC.[SA], DC.[SR], DC.[SD], DC.[SE], DC.[SI], DC.[SG], DC.[SN], DC.[SL], DC.[SK], DC.[SP], DC.[SS], DC.[ST], DC.[SV], DC.[TA], DC.[TR], DC.[TD], DC.[TE], DC.[TI], DC.[TG], DC.[TL], DC.[TP], DC.[TS], DC.[TT], DC.[TV], DC.[YG], DC.[YL], DC.[VA], DC.[VR], DC.[VD], DC.[VE], DC.[VI], DC.[VG], DC.[VN], DC.[VL], DC.[VK], DC.[VP], DC.[VS], DC.[VT], DC.[VV], MTF.[EHH], MTF.[LS-GR], MTF.[S-G-G-R], PCP.[Solvent contact area], PCP.[Flexibility]. (Total number: 174) |
| Eukaryota | AAC.[A], AAC.[R], AAC.[C], AAC.[L], AAC.[K], AAC.[S], DC.[AA], DC.[AR], DC.[AD], DC.[AE], DC.[AI], DC.[AG], DC.[AN], DC.[AL], DC.[AK], DC.[AF], DC.[AP], DC.[AS], DC.[AT], DC.[AV], DC.[RA], DC.[RR], DC.[RC], DC.[RG], DC.[RN], DC.[RL], DC.[RK], DC.[RS], DC.[RV], DC.[DA], DC.[DR], DC.[DD], DC.[DE], DC.[DI], DC.[DG], DC.[DL], DC.[DK], DC.[DP], DC.[DT], DC.[DV], DC.[CA], DC.[CC], DC.[CQ], DC.[CE], DC.[CI], DC.[CK], DC.[CP], DC.[CS], DC.[CT], DC.[QA], DC.[QE], DC.[QN], DC.[QL], DC.[QK], DC.[QV], DC.[EA], DC.[ER], DC.[ED], DC.[EE], DC.[EG], DC.[EN], DC.[EL], DC.[EK], DC.[EF], DC.[ES], DC.[ET], DC.[EV], DC.[IA], DC.[IR], DC.[ID], DC.[IQ], DC.[IE], DC.[II], DC.[IG], DC.[IL], DC.[IK], DC.[IF], DC.[IP], DC.[IS], DC.[IT], DC.[IV], DC.[GA], DC.[GR], DC.[GD], DC.[GC], DC.[GE], DC.[GI], DC.[GG], DC.[GN], DC.[GL], DC.[GK], DC.[GP], DC.[GS], DC.[GT], DC.[GY], DC.[GV], DC.[NC], DC.[NI], DC.[NG], DC.[NN], DC.[NL], DC.[NK], DC.[NF], DC.[NP], DC.[NV], DC.[LA], DC.[LR], DC.[LD], DC.[LC], DC.[LQ], DC.[LE], DC.[LI], DC.[LG], DC.[LN], DC.[LL], DC.[LK], DC.[LP], DC.[LS], DC.[LT], DC.[LV], DC.[KA], DC.[KR], DC.[KD], DC.[KC], DC.[KQ], DC.[KE], DC.[KI], DC.[KG], DC.[KN], DC.[KL], DC.[KK], DC.[KS], DC.[KT], DC.[KY], DC.[KV], DC.[MA], DC.[MK], DC.[FA], DC.[FD], DC.[FL], DC.[FK], DC.[FS], DC.[FV], DC.[PA], DC.[PR], DC.[PD], DC.[PE], DC.[PG], DC.[PL], DC.[PK], DC.[PP], DC.[PS], DC.[PV], DC.[SA], DC.[SR], DC.[SD], DC.[SC], DC.[SE], DC.[SI], DC.[SG], DC.[SN], DC.[SL], DC.[SK], DC.[SF], DC.[SP], DC.[SS], DC.[ST], DC.[SV], DC.[TA], DC.[TC], DC.[TE], DC.[TI], DC.[TG], DC.[TL], DC.[TK], DC.[TP], DC.[TS], DC.[TT], DC.[TV], DC.[YL], DC.[YS], DC.[VA], DC.[VR], DC.[VD], DC.[VE], DC.[VI], DC.[VG], DC.[VN], DC.[VL], DC.[VK], DC.[VF], DC.[VP], DC.[VS], DC.[VT], DC.[VV], MTF.[GM-E], MTF.[FVE], MTF.[TGD], MTF.[F-YG], PCP.[Hydrophilicity], PCP.[Polarity], PCP.[Transfer free energy], PCP.[Positively charge], PCP.[Flexibility]. (Total number: 204) |
| Archaea | AAC.[A], AAC.[R], AAC.[D], AAC.[E], AAC.[I], AAC.[K], DC.[AA], DC.[AR], DC.[AD], DC.[AE], DC.[AI], DC.[AG], DC.[AL], DC.[AK], DC.[AS], DC.[AT], DC.[AV], DC.[RA], DC.[RR], DC.[RD], DC.[RC], DC.[RE], DC.[RI], DC.[RG], DC.[RL], DC.[RP], DC.[RV], DC.[DA], DC.[DR], DC.[DD], DC.[DE], DC.[DG], DC.[DL], DC.[DP], DC.[DS], DC.[DV], DC.[CG], DC.[QF], DC.[EA], DC.[ER], DC.[ED], DC.[EE], DC.[EI], DC.[EG], DC.[EL], DC.[EK], DC.[ES], DC.[ET], DC.[EY], DC.[EV], DC.[IA], DC.[IR], DC.[ID], DC.[IE], DC.[II], DC.[IG], DC.[IN], DC.[IL], DC.[IK], DC.[IS], DC.[IV], DC.[GA], DC.[GR], DC.[GD], DC.[GE], DC.[GI], DC.[GG], DC.[GL], DC.[GK], DC.[GS], DC.[GT], DC.[GV], DC.[NV], DC.[LA], DC.[LR], DC.[LD], DC.[LE], DC.[LI], DC.[LG], DC.[LN], DC.[LL], DC.[LK], DC.[LP], DC.[LS], DC.[LV], DC.[KA], DC.[KR], DC.[KE], DC.[KI], DC.[KG], DC.[KN], DC.[KL], DC.[KK], DC.[KT], DC.[KV], DC.[MV], DC.[FA], DC.[FD], DC.[FE], DC.[PR], DC.[PD], DC.[PE], DC.[SA], DC.[SE], DC.[SI], DC.[SG], DC.[SL], DC.[SK], DC.[TA], DC.[TD], DC.[TL], DC.[TS], DC.[YK], DC.[VA], DC.[VR], DC.[VE], DC.[VI], DC.[VG], DC.[VL], DC.[VK], DC.[VV], MTF.[G-GW], MTF.[A-TLD], MTF.[A-T-LD], MTF.[DG-W], PCP.[Hydrophobicity], PCP.[Polarity], PCP.[Positively charge], PCP.[Flexibility]. (Total number: 129) |

*Red, black, blue and orange words indicate AAC-, DC-, MTF- and PCP-type features respectively.*


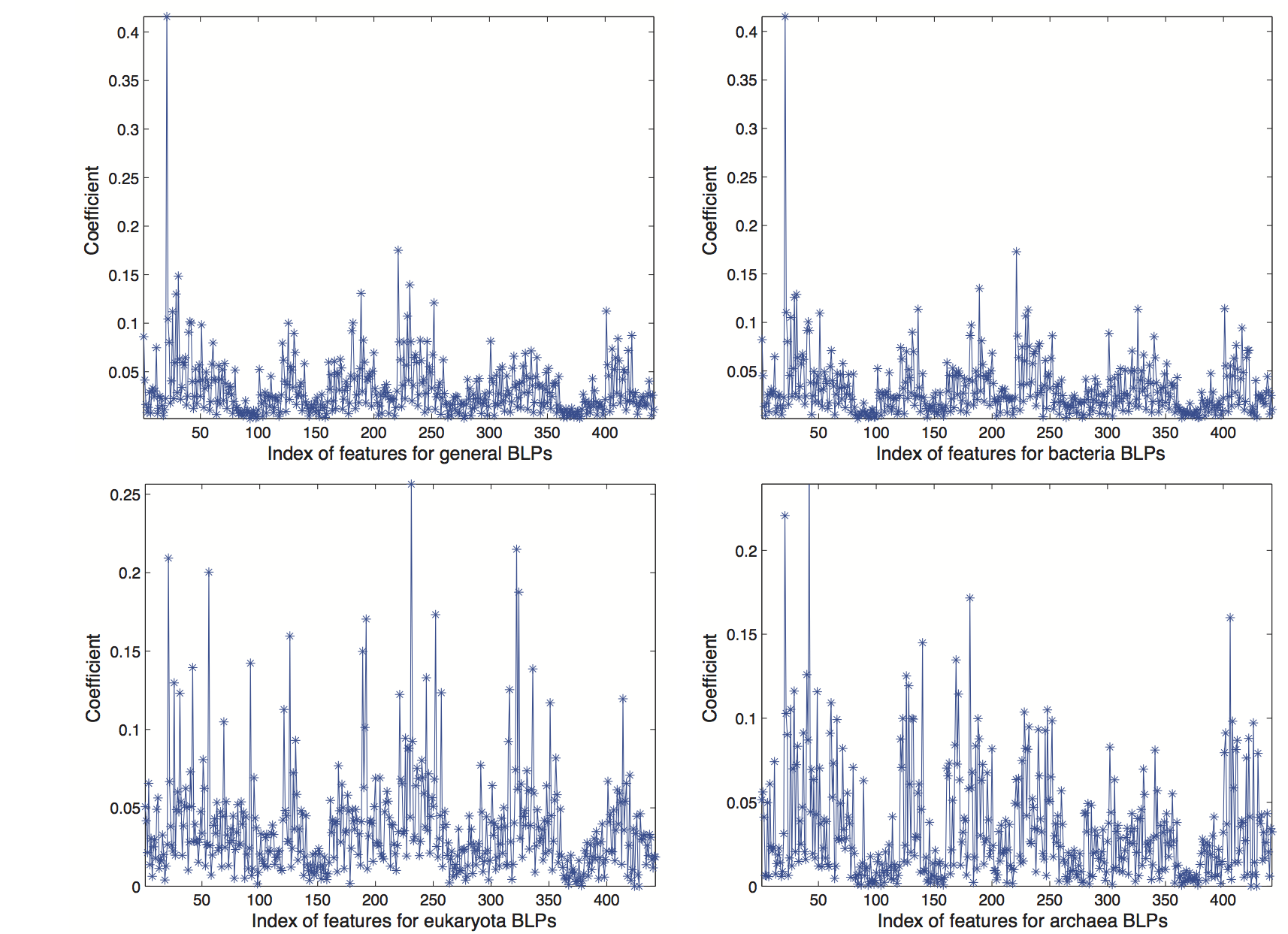


**Additional Figure A1** The calculated correlation coefficient between labels and each feature in four training datasets.


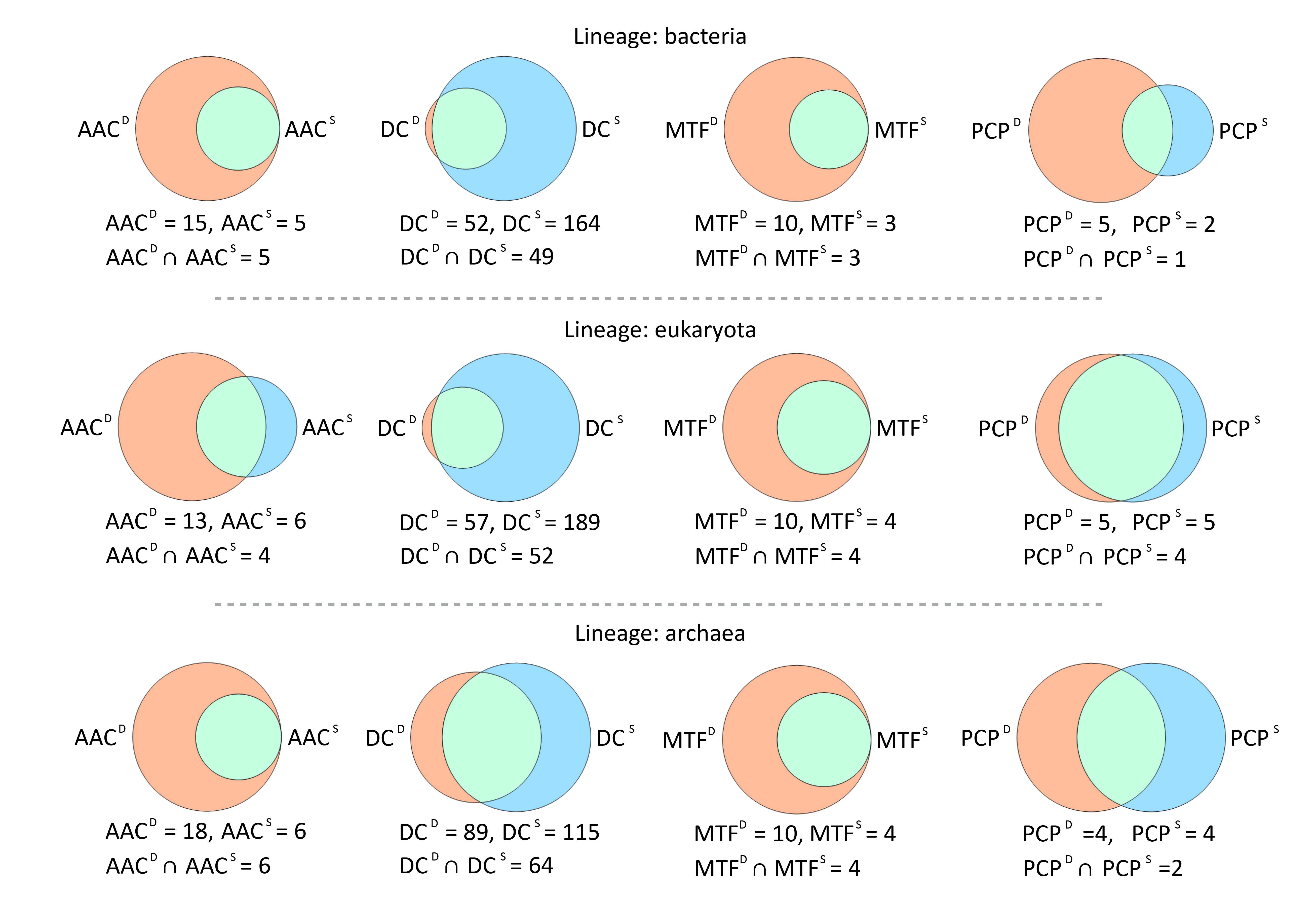


**Additional Figure A2** Venn diagrams of the overlap between the discriminatory and selected useful features in the optimal subset for each type of features.

^D^ indicates the discriminatory features and ^S^ stands for selected useful features.
